# Effect of Retirement on Cognition: Evidence From the Irish Marriage Bar

**DOI:** 10.1007/s13524-018-0682-7

**Published:** 2018-06-07

**Authors:** Irene Mosca, Robert E. Wright

**Affiliations:** 1 The Irish Longitudinal Study of Ageing (TILDA), Lincoln Gate, Trinity College Dublin, Dublin 2, Republic of Ireland; 2 Department of Economics, University of Strathclyde, 199 Cathedral Street, Glasgow, UK

**Keywords:** Cognition, Aging, Working, Retirement, Women

## Abstract

This study empirically investigates the relationship between retirement duration and cognition among older Irish women using microdata collected in the third wave of The Irish Longitudinal Study on Ageing. Ordinary least squares (OLS) regression estimates indicate that the longer an individual has been retired, the lower the cognitive functioning, with other factors thought to affect cognition held constant (e.g., age, education, and early-life socioeconomic conditions). However, retirement is potentially endogenous with respect to cognition because cognition may affect decisions relating to retiring. If so, the OLS estimates will be biased. To test for this possibility, instrumental variable (IV) estimation is used. This method requires an IV that is highly correlated with retirement duration but not correlated with cognition. The instrument used in this study is based on the so-called marriage bar, the legal requirement that women leave paid employment upon getting married, which took effect in Ireland in the 1930s and was abolished only in the 1970s. The IV regression estimates, along with formal statistical tests, provide no evidence in support of the view that cognition affects retirement decisions. The finding of a small negative effect of retirement duration on cognition is robust to alternative empirical specifications. These findings are discussed in the wider context of the effects of work-like and work-related activities on cognition.

**Electronic supplementary material:**

The online version of this article (10.1007/s13524-018-0682-7) contains supplementary material, which is available to authorized users.

## Introduction

Many cognitive abilities decline in old age, and age-related declines in cognitive abilities are correlated with declines in the ability to perform everyday tasks (Tucker-Drob 2011). Researchers, however, are showing greater recognition of individual differences in the extent to which cognitive abilities are lost or maintained in old age (Tucker-Drob and Salthouse 2011). Two questions are of particular importance. First, Who are the people who are able to maintain cognitive abilities (e.g., memory, reasoning, and processing speed) in old age? Second, How do these people differ from those who cannot maintain cognitive abilities in old age?

Two main (and somewhat competing) hypotheses have been used to explain observed heterogeneity across individuals with respect to the decline in or preservation of cognitive abilities in old age. The *cognitive reserve hypothesis* suggests that the advantages afforded by early-life socioeconomic opportunities serve to slow the rate of age-related cognitive decline (Stern 2002, 2003). Individuals who experience more enriched socioeconomic environments during childhood and early adulthood have more resilient cognitive and/or neurobiological architectures in adulthood and, in turn, experience less cognitive decline as they age. Research suggests that one of the best indicators of socioeconomic advantages is educational attainment (Tucker-Drob and Salthouse 2011).

The *mental exercise hypothesis*—also known as *cognitive enrichment* or *cognitive use hypothesis* (Hertzog et al. 2008; Hultsch et al. 1999; Salthouse 2006)—suggests that individuals who engage in mental exercise and maintain an engaged lifestyle experience relatively less cognitive decline. More specifically, it is argued that high levels of neuronal activation brought about by mental stimulation can buffer against neuro-degeneration and cognitive decline in old age (Churchill et al. 2002; van Praag et al. 2000). An early proponent of this position (Sorenson 1938) suggested that to prevent cognitive decline, people should order their lives such that they constantly find themselves in new situations and confronted with novel problems. The view that keeping mentally active will maintain one’s level of cognitive functioning—and possibly prevent cognitive decline—is so pervasive in contemporary culture that it is frequently expressed in curt terms: “use it or lose it” (Salthouse 2006:70).

If engaging in mental exercise can indeed help maintain cognitive functioning and possibly prevent cognitive decline (as suggested by the mental exercise hypothesis), the next logical question is, Which types of activities are most beneficial? Mentally stimulating activities hypothesized as protective against age-related declines in cognition include recreational activities, such as doing crossword puzzles and playing chess, or learning a new skill or how to speak a foreign language (Tucker-Drob and Salthouse 2011). A small albeit growing body of research has suggested that another way to preserve cognition is to delay retirement and continue to work into the later years. The hypothesis is that workers engage in more mental exercise than retirees because work environments provide more cognitively challenging and stimulating environments than do nonwork environments. Thus, perhaps a negative relationship exists between retirement and cognitive functioning (cognition).

Our study adds to the small but growing body of research that empirically tests the validity of this specific instantiation of the “use it or lose it” hypothesis, using data from Ireland. The relationship between retirement and cognitive functioning is investigated using data for older Irish women collected in the third wave of The Irish Longitudinal Study on Ageing (TILDA). Ordinary least square (OLS) regressions are used in first instance. Because retirement is potentially endogenous with respect to cognition, instrumental variable (IV) estimation is also used. The identifying instrument in the IV estimation is the abolition of the so-called marriage bar, which was the legal requirement that women leave paid employment on getting married.

Our analysis suggests a negative effect of retirement duration on cognitive functioning. That is, the longer an individual has been retired, the lower the cognitive functioning, holding other factors constant with multiple regression. However, this effect is small. We see no evidence that retirement is endogenous, in the sense that there is no evidence that cognitive functioning has an effect on retirement duration. The findings are found to be robust to alternative empirical specifications.

## Contribution to the Literature

Several recent studies in the fields of both health and economics have investigated the effect of retirement on cognition. However, the focus is somewhat different between the two sets of studies. Health-based studies have mainly used longitudinal data and methods to investigate the effect of retirement on cognitive decline, concerned primarily with intraindividual changes in cognitive functioning over time. Most of these studies explored whether the nature of employment in the preretirement occupation affects the rate of cognitive decline after retirement. On the other hand, economics-based studies have argued that the main challenge to identifying the effect of retirement on cognition is that the decision to retire might itself be affected by cognition (Rohwedder and Willis 2010). In other words, the direction of causation between retirement duration and cognition may be two-way. If this were the case, the key empirical challenge is to determine which causal direction dominates. Most of the economics-based studies have used a statistical technique known as the *instrumental variable estimation* to address this issue. We describe the technique in detail later.

The first study to investigate the effect of retirement on cognitive decline in an epidemiological sample was Roberts et al. (2011). Using data spanning a five-year period from the UK Whitehall II Study, they found that individuals who retired in the study period showed a trend toward smaller cognitive test score increases than those who were still working at follow-up. Using data spanning a six-year period from the Swedish National Study on Aging and Care, Rennemark and Berglund (2014) found that participants who retired prior to age 60 experienced cognitive decline in the study period. Cognitive decline was not found for those who worked in the study period.

Finkel et al. (2009), Fisher et al. (2014), and Andel et al. (2015) employed latent growth models to investigate whether job characteristics during one’s time of employment moderate the association between retirement and cognitive decline. Using data from a subset of twins from the population-based Swedish Twin Registry, Finkel et al. (2009) found larger negative effects of retirement on cognitive decline for individuals whose preretirement jobs were characterized by high levels of “complexity” for some (but not all) measures of cognition included in their data set. Fisher et al. (2014), using longitudinal data spanning 18 years from the U.S. Health and Retirement Study (HRS), found that individuals with preretirement jobs that were characterized by higher “mental demands” had less steep cognitive declines after retirement. Likewise, Andel et al. (2015), using multiple waves of the HRS, found that individuals whose preretirement jobs were characterized by “less control” and “greater strain” had steeper cognitive declines after retirement.

To our knowledge, only five economics-based studies have investigated the effect of retirement on cognition. Four of these studies used IV estimation to explore the endogeneity of retirement. The IV approach requires a variable (instrument) that is correlated with the retirement decision but not correlated with cognition. It also needs to be exogenous in the sense that it is not a direct outcome of individual decision-making.

Using data collected in the HRS, the English Longitudinal Study on Ageing (ELSA) and the multicountry Survey of Health, Retirement and Ageing in Europe (SHARE), Rohwedder and Willis (2010) and Mazzonna and Peracchi (2012) employed cross-country and temporal changes in policies affecting the age at which individuals are entitled to receive a state-supplied pension and other age-related benefits. The expectation is that this variability would have a sizable effect on retirement decisions but have no direct effect on cognition. Before and after controlling for endogeneity, both studies found sizable negative effects of retirement on cognition. Bonsang et al. (2012), using data from the HRS, reached a similar conclusion following a similar approach. de Grip et al. (2015), using Dutch data from the Maastricht Aging Study, found large negative effects of retirement on cognitive decline for some (but not all) measures of cognition included in their data set. Finally, Coe et al. (2012), also using HRS data, used early retirement offers (which are legally required to be nondiscriminatory) as a source of exogenous variation, and found no support that retirement affects cognition.

Our study differs from the previous studies in three main ways. First, our analysis focuses on women. The employment histories for men and women are generally different. In most high-income countries, men typically work uninterruptedly from when they complete schooling until retirement, with ill health and unemployment being the main factors causing deviation from this pattern. The pattern for women is typically different because childbearing and child-rearing frequently result in mothers leaving the labor force, often for considerable periods of time. With the exception of Mazzonna and Peracchi (2012), the existing studies focused only on men or did not disaggregate the analysis by sex. Grouping men and women may mask important differences. For all these reasons, we believe it important to analyze women separately—and even more important, not to exclude them.

Second, the differences in the findings of the economics-based studies may be a product of differences in the exogenous variation used in the statistical models. Basically, this variation is caused by policy changes that should affect retirement decisions. However, it assumes that individuals are rational and fully understand these changes. Considerable evidence shows that this is not the case (see, e.g., Hancock et al. 2004). Therefore, we exploit an alternative source of exogenous variation unique to the Irish context caused by the abolition of the so-called marriage bar. The marriage bar was the legal requirement that women leave paid employment—in a sense, retire from paid work—upon marrying. It was established in the 1930s and abolished in the 1970s. The TILDA data used here surveyed women who were required to leave paid employment—retire—because of the marriage bar. Many of these women spent a significant proportion of their lives after getting married in retirement.

Third, the TILDA data include measures of cognition that are novel in the context of other large-scale, nationally representative studies on aging. One unique feature is that they are administered and scored by nurses trained specifically for this purpose. Therefore, they should be subject to less measurement error compared with self-assessed or interviewer-administered measures. The four measures of cognition employed in the analysis of our study capture processing speed and mental switching, which are central to effective cognitive functioning. Crucially, both processing speed and mental switching require effortful processing at the time of assessment and do not require production of previously acquired knowledge (Tucker-Drob and Salthouse 2011).

## Methodology

### Data

The data we use are from the third wave of TILDA, which is a nationally representative sample of community-dwelling individuals aged 50 or older in Ireland. The survey collects detailed information on the economic, health, and social aspects of the respondents’ lives. It is modeled closely on HRS, ELSA, and SHARE. At the Wave 3 interview (2014/2015), 6,566 respondents completed a computer-assisted personal interview (CAPI) in their homes and were invited to travel to a dedicated health center based in Trinity College Dublin for a comprehensive health assessment. If unable or unwilling to travel to the health center, respondents were offered a modified assessment in their home. All assessments were carried out by qualified and trained research nurses. A total of 5,395 respondents underwent a health assessment: 80 % in the Trinity College Dublin health center and 20 % in their home. Although the main analysis of this article is based on data from the third wave of TILDA, data on labor market circumstances from the first (2009/2011) and second (2012/2013) waves were also employed to construct the relevant labor market variables or for robustness checks. For more detail about TILDA, see Cronin et al. (2013), Kearney et al. (2011), and Whelan and Savva (2013).

### Statistical Model

In our statistical model, we assume that cognition (*Cog*) is a function of retirement duration (*RetDur*), a vector of other controls (**X**_*j*_) (such as *j* = age and education), and an error term (*u*). In regression form,

1 $$ {Cog}_i={\upbeta}_0+{\upbeta}_1{RetDur}_i+{\sum}_j{\upbeta}_j{\mathbf{X}}_{ij}+{u}_i, $$

where the subscript *i* denotes the individual, *i* = 1, 2, . . . , *N*. If *RetDur* is correlated with *u*, then OLS estimates of β_1_ will be biased and inconsistent. IV estimation can be used to purge the relationship between *RetDur* and *Cog* of this bias. Key to IV estimation is the availability of at least one variable, *Z* (instrument), which has the following three key properties: (1) variation in *Z* is associated with variation in *RetDur*; (2) variation in *Z* is not associated with variation in *Cog* (apart from the indirect route via *RetDur*); and (3) variation in *Z* is not associated with variation in unmeasured variables that affect *RetDur* and *Cog*. If one has available a variable that satisfies these properties, then one can estimate the following regression:

2 $$ {RetDur}_i={\uppi}_0+{\uppi}_1{Z}_i+{\sum}_j{\uppi}_j{\mathbf{X}}_{ij}+{w}_i, $$

where *RetDur* is as a function of *Z*, **X**_*j*_, and an error term *w*. By estimating this first-stage regression, one can then form predictions for *RetDur*:

3 $$ {\widehat{RetDur}}_i=\widehat{\uppi_0}+\widehat{\uppi_1}{Z}_i+{\sum}_j\widehat{\uppi_j}{\mathbf{X}}_{ij}. $$

One can use OLS to estimate the second-stage regression:

4 $$ {Cog}_i={b}_0+{b}_1{\widehat{RetDur}}_i+{\sum}_j{b}_j{\mathbf{X}}_{ij}+{e}_i, $$

where predicted values of *RetDur* from Eq. (3) are used. Assuming that all assumptions are met, the error term in this regression, *e*, is random and not correlated with *RetDur*. If this is the case, Eq. (4) will provide an unbiased estimate, *b*_1_, of the relationship between retirement duration and cognition. On the other hand, if *b*_1_ = β_1_ (which is a testable hypothesis), retirement duration is exogenous, and OLS provides such an estimate.

A note of caution is needed when using IV estimation. For all analyses using IV estimation, generalizability is a concern because the IV estimation recovers what in the literature is referred to as the *local average treatment effect* (LATE) (Angrist and Imbens 1994). The LATE is the average effect of the treatment among only the group affected by the instrument. In our analysis, IV estimates the average effect of retirement duration on cognition for the group of women who were affected by the instrument *Marriage Bar* because the law was in place but would not have not been affected had the law not been in place.

### Variables

#### Cognition

The four cognition variables are tests of processing speed and mental switching that have been widely used and validated in clinical studies. The Colour Trail Task 1 test (CTT1) captures mainly visual scanning and mental processing speed. The Colour Trail Task 2 test (CTT2) captures additional executive functions, such as task switching (D’Elia et al. 1996). The Choice Reaction Time (CRT) and Choice Reaction Time Variability (CRT_VAR) tests capture processing speed and concentration. Importantly, these tests require effortful processing at the time of assessment and do not require production of previously acquired knowledge (Tucker-Drob and Salthouse 2011).

In TILDA, cognitive tests are administered and scored by trained and qualified nurses during the health assessment. Focusing on the four tests employed in this study, respondents are first passed a sheet of paper containing numbers in yellow or pink circles. For the CTT1, respondents are instructed to rapidly draw a line with a pencil, connecting the circles numbered 1–25 in consecutive order. In the CTT2, respondents are asked to connect numbered circles alternating between pink and yellow circles (e.g., pink 1, yellow 2, pink 3, and so on). The performance indicator for both CTT1 and CTT2 is the time taken (in seconds) to successfully complete the test, with shorter completion times indicative of better performance.

Respondents are then required to perform a computer-based task. They are asked to depress a central button until a stimulus appears on-screen: either the word YES or the word NO. Each time a stimulus appears, respondents are required to press the corresponding button. A return to the central button is necessary after each response for the next word to appear on-screen. There are approximately 100 repetitions. The task variables of interest are the mean intraindividual CRT and the standard deviation of individual CRT, the latter providing a measure of variability (CRT_VAR). CRT and CRT_VAR are measured in milliseconds.

**Fig. 1:**
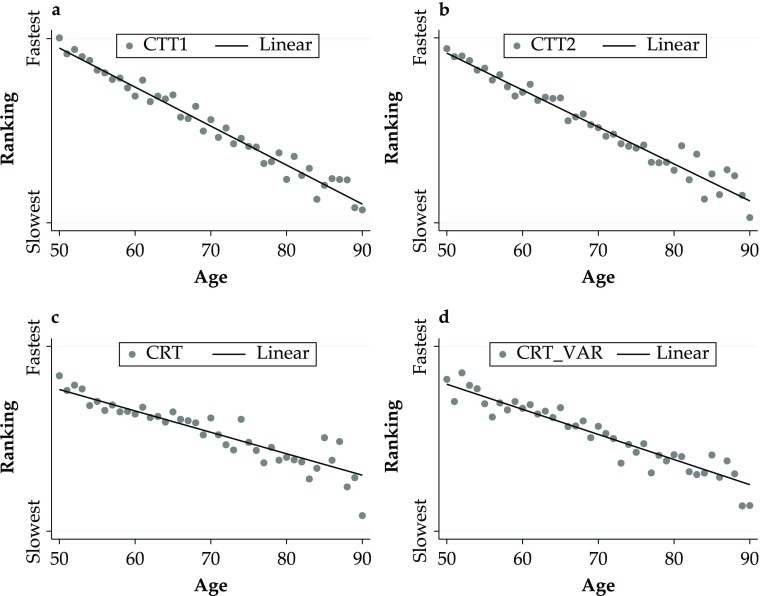
Cognitive measures by age. Panel a shows Colour Trail Task 1 (CTT1). Panel b shows Colour Trail Task 2 (CTT2). Panel c shows Choice Reation Time (CRT). Panel d shows Choice Reation Time Variabiliy (CRT_VAR)

In Fig. 1, panels a–d plot the relationship between age and the four cognition measures. For each measure, respondents were ranked from slowest to fastest based on the time taken to complete the task. Then the mean ranking position by year of age was computed for each of the four cognitive measures. Figure 1 shows a clear negative relationship between age and cognition. For completeness, the relationship between age and the four cognitive measures expressed in the original metric (i.e., time taken to complete the task) is illustrated in Fig. S1 in Online Resource 1. The relationship between age and the standardized values (*z* scores) of the cognition variables is also shown in the same figures.

#### Retirement Duration

In the CAPI interview, respondents are asked to report the status that best describes their current labor market situation: (1) retired, (2) employed, (3) self-employed, (4) unemployed, (5) permanently sick or disabled, (6) looking after home or family, (7) in education or training, and (8) other. Respondents can select only one choice because the options are designed to be mutually exclusive. At the Wave 3 interview, 34.5 % of women in the sample are employed or self-employed, and another 40.5 % are retired. Nearly one-fifth (19.4 %) are looking after home or family; 3.1 % are permanently sick or disabled, and 2 % are unemployed. We classify an individual as *working* if she reports to be currently in employment, or *retired* otherwise. Working individuals are, therefore, those who chose categories (2) and (3), and retired individuals are those who chose categories (1) and (4)–(8). Robustness checks concerned with the reliability of our definition of retirement are reported later herein.

Respondents not working at the time of the interview are then asked whether they have done any paid work in the week prior to the interview. Individuals who reported to have done some paid work in that week (*n* = 56) are excluded. A total of 160 respondents reported to have never done any paid work. Some of these respondents may have engaged in unpaid work at some point over their lifetime—for example, on the family farm or in the family business. Unfortunately, additional information on the employment history of respondents who report never having done any paid work is not collected in TILDA. For this reason, these respondents are excluded from the analysis. Only respondents who report having done paid work at some point in their life are kept in the sample.

Respondents in categories (1) and (4)–(8) are asked to report the month and year when they stopped working. For example, respondents who report being retired (i.e., in category (1)) are asked the following question: “In what month/year did you stop working?” Similarly, respondents who report being unemployed (i.e., in category (4)) are asked the following question: “In what month/year did you become unemployed?” We define *retirement duration* as the time elapsed between the date the respondent stopped working and the date of the health assessment for that respondent. Retirement duration in full months is calculated and converted to years of retirement for ease of interpretation. For those at work, retirement duration is set to 0.

Because information on labor market status is also collected at Waves 1 and 2 with the same questions, this information is used to construct a more robust measure of retirement duration. If inconsistent answers are provided across the three waves, we consider as most reliable the measure of retirement duration constructed based on Wave 1 reports, followed by Wave 2 reports and Wave 3 reports. This should minimize recall bias: the time elapsed between the date of retirement and the date of interview is shorter because Wave 1 occurs before Waves 2 and 3. Retirement duration cannot be calculated for 117 women because of missing information, and these individuals are excluded from the sample.

**Fig. 2:**
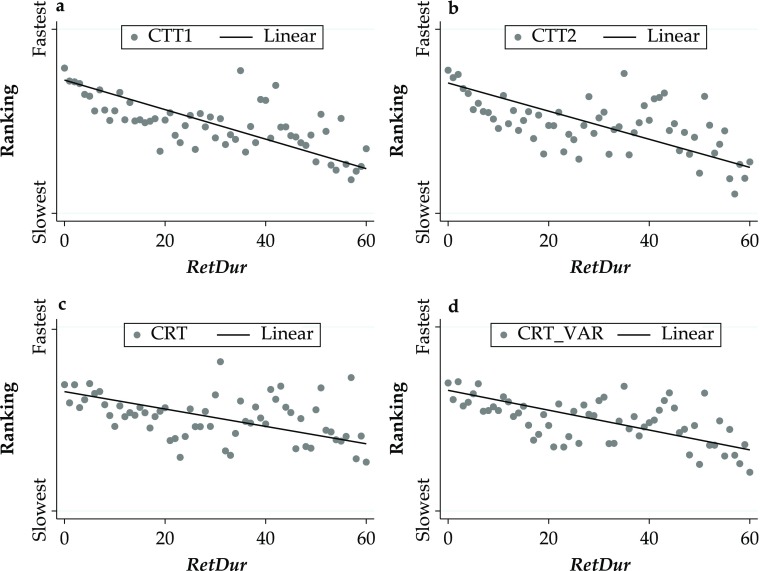
Cognitive measures by retirement duration. Panel a shows Colour Trail Task 1 (CTT1). Panel b shows Colour Trail Task 2 (CTT2). Panel c shows Choice Reation Time (CRT). Panel d shows Choice Reation Time Variabiliy (CRT_VAR)

Panels a–d of Fig. 2 plot the relationship between retirement duration and the four cognitive measures, showing that respondents who have retired for longer are, on average, slower at completing the cognition tasks. The relationship between retirement duration and the four cognitive measures expressed in the original time metric and between age and the standardized values (*z* scores) of the cognition variables is shown in Fig. S2 in Online Resource 1.

#### Controls

Additional variables thought to affect cognition are included. These variables include the key factors of age and education, as well as a set of variables aimed at capturing childhood characteristics. The main aim is to restrict the list of control variables to those that are clearly exogenous and not subject to same endogeneity considerations as retirement duration. We achieve this aim by selecting variables measured when the respondent was young.

The relationship between education and cognition has been studied. A number of studies have found evidence that education positively affects cognition in later life (e.g., Banks and Mazzonna 2012; Schneeweis et al. 2014). Because most schooling among older Irish women is completed when they are young and before they enter the labor market, it is exogenous. Education (*School*) is measured as the number of years of schooling completed.

Several childhood characteristics have been shown to be associated with cognition in later life (Borenstein et al. 2006; Brown 2010; Everson-Rose et al. 2003). We employ a set of dummy variables based on respondent’s self-reporting of childhood conditions before age 14: *NoBook* = 1 if there were no or very few books in the home where respondent grew up (0 = otherwise); *PoorHealth* = 1 if respondent was in fair/poor health (0 = otherwise); *PoorFam* = 1 if respondent grew up in a poor family (0 = otherwise); *MotherNotWork* = 1 if respondent’s mother never worked outside the home (0 = otherwise); and *FatherNotWork* = 1 if respondent’s father never worked outside the home (0 = otherwise). For 37 women, information is missing on one or more of these variables, and these individuals are excluded from the sample.

The final samples are 2,519 women for the model based on CTT1; 2,481 women for the model based on CTT2; and 2,383 women for the models based on CRT and CRT_VAR. Table 1 displays descriptive statistics for all independent variables based on the sample including 2,519 women. The average age is 65.8 years, and the average retirement duration is 12 years.

**Table 1:** Means and standard deviations of regression independent variables

Variable	Definition	Measurement	Mean	SD
*Age*	Age of respondent	Years	65.8	9.0
*RetDur*	Retirement duration, defined as time elapsed since last job ended	Years	12.0	15.7
*School*	Schooling	Years completed	12.4	2.7
*NoBooks* (%)	Books where respondent lived in childhood	Dummy variable: 1 for 0–10 books; 0 for 11+ books	34.0	––
*PoorHealth* (%)	Self-reported health in childhood	Dummy variable: 1 for poor/fair; 0 for excellent/very good/good	6.1	––
*PoorFam* (%)	Self-reported socioeconomic position in childhood	Dummy variable: 1 for poor; 0 for average/well-off	15.4	––
*MotherNotWork* (%)	Mother ever worked outside the home in childhood	Dummy variable: 1 for mother never worked; 0 otherwise	70.1	––
*FatherNotWork* (%)	Father ever worked outside the home in childhood	Dummy variable: 1 for father never worked; 0 otherwise	6.5	––

#### Instrumental Variable: The Marriage Bar

We believe that the abolition of the so-called marriage bar in Ireland caused exogenous variation in retirement decisions. The marriage bar was the legal requirement that women leave their paid employment after getting married. It was established for primary school teachers in 1933 and for civil servants in 1956. Although not legally obliged to do so, many semi-state and private organizations—including banks, utility companies, and large manufacturers—also dismissed women when they married. Private sector employers dismissed women working in primarily clerical and skilled jobs, but in some cases, they dismissed unskilled workers (Kiely and Leane 2012:91).

The marriage bar for primary school teachers was lifted in 1958, and lifted for civil servants in 1973. Discrimination in employment on the grounds of sex or marital status was made illegal in 1977. Unsurprisingly, the labor force participation rate of married women aged 15 and older increased from 7.5 % in 1971 to 14.5 % in 1975 (Pyle 1990). For more on the Irish marriage bar, see Connolly (2003), Cullen Owens (2005), Kiely and Leane (2012), and O’Connor (1998).

**Fig. 3:**
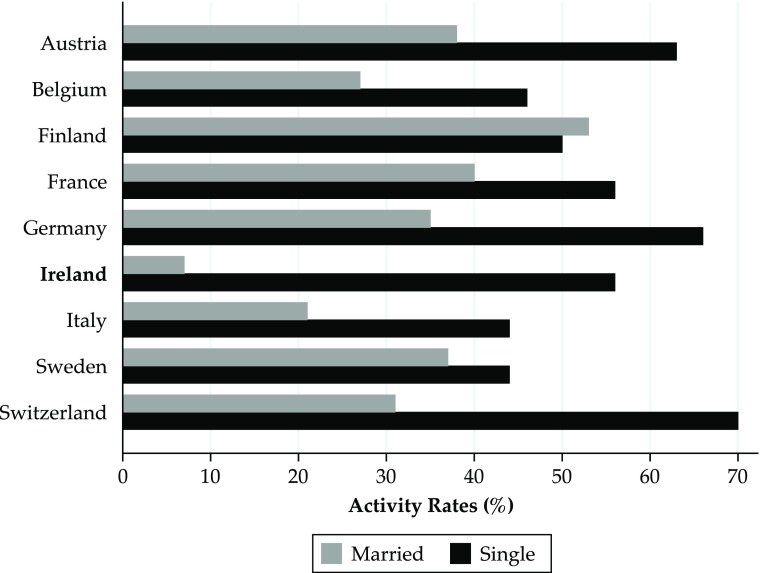
Activity rates (%) by marital status for women aged 15+: Various countries, 1970. *Source:* Pyle (1990)

Crucially, no evidence exists that the marriage bar forced women to choose between paid employment or getting married. For example, Fig. 3 shows female activity rates for married and single women in 1970 in Ireland and other countries. Clearly, although activity rates of single women in Ireland were closely aligned to activity rates of single women in other countries, married women in Ireland were significantly less likely to be active than those in other countries. This suggests that an exogenous factor preventing married women from working in Ireland was present, which we believe is the marriage bar.

**Fig. 4:**
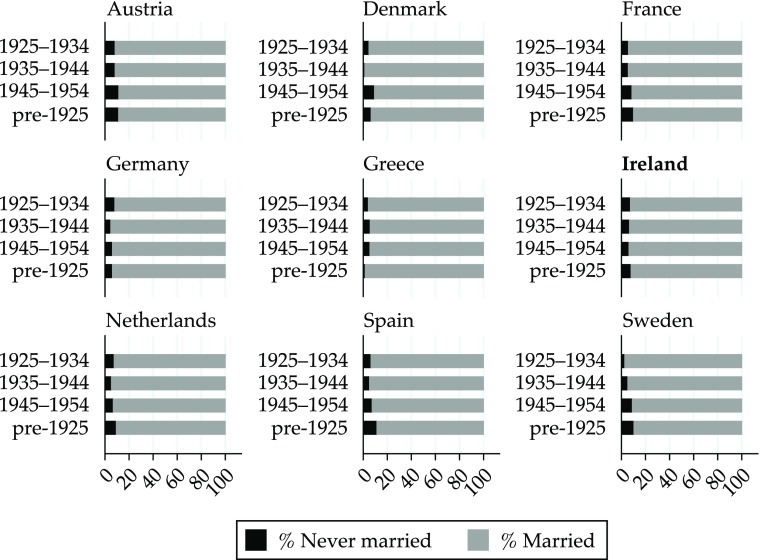
Proportion of never-married and married women by birth cohort: Various countries. *Source:* Authors’ calculations from SHARE and TILDA

**Fig. 5:**
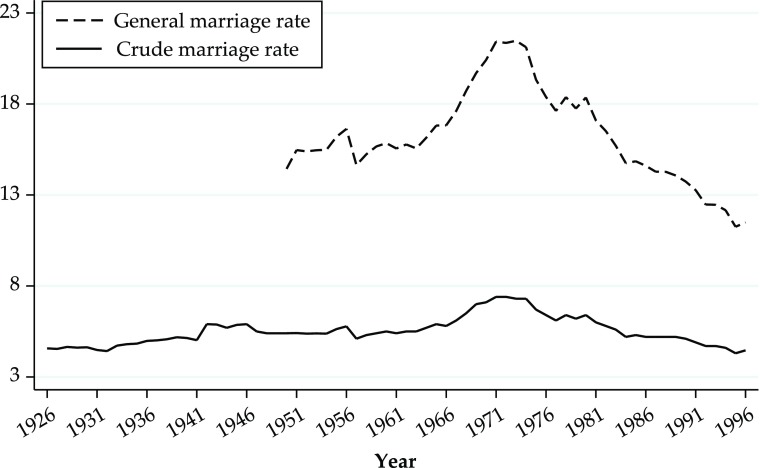
Crude and general marriage rate (MR): Ireland, 1926–1996. General MR is defined as number of marriages per 1,000 female population aged 15+. Crude MR is defined as number of marriages per 1,000 population. *Source:* Central Statistics Office (1926–2000)

Additional evidence consistent with this view is shown in Figs. 4 and 5. Figure 4 shows the proportions of never-married and married women calculated from the TILDA and SHARE surveys by birth cohort. In Ireland, like in many other countries, the proportion of never-married women is very small, suggesting that marriage was the norm for women born in the first half of the twentieth century. Figure 5 shows the historical crude marriage rate and the general marriage rate for Ireland (1926–1996). One would expect that if women were forced to choose between marriage and paid employment, the marriage rate would increase after the abolition of the marriage bar. Figure 5 shows that, if anything, the marriage rate stabilized and then decreased after the abolition of the marriage bar: that is, it moved in the opposite direction.

Ireland is not the only country where women were dismissed from employment at marriage. For example, marriage bars survived up to the 1950s in the United States (Goldin 1990), England (Smith 1986), the Netherlands (Boeri and van Ours 2013), and Germany (Kolinsky 1989). Ireland is, however, unique in the duration of the enforcement of the marriage bar. Many Irish women who were affected are still alive and are in the TILDA sample. Comparatively, most of the women affected by the marriage bar in the other countries are likely to have died or to be very old.

TILDA is the first large-scale longitudinal study on aging to include specific questions on the marriage bar. In TILDA Wave 3, women are asked the following question: “Did you ever have to leave a job because of the Marriage Bar?” The instrument used is a dummy variable, *MarBar*, coded 1 if a woman reported having to leave employment on getting married and 0 otherwise. It is also coded 0 for the few women in the sample who reported never marrying. Of the 2,519 women in the final sample, 318 reported that they had to leave a job because of the marriage bar. Some of these women subsequently returned to work. For these women, the instrument is coded 1, and *RetDur* is defined as the time elapsed between the date the respondent stopped working in her final job and the date of the health assessment for that respondent.

## Results

### Main Empirical Findings

Columns 1 and 2 in Tables 2 and 3 show the OLS regression estimates for CTT1 and CTT2, and CRT and CRT_VAR, respectively. We transform the four outcome variables by taking the natural logarithm in order to ensure normality of the residuals. We then multiply the transformed scores by –1. Therefore, a higher value of these transformed variables suggests a higher level of cognitive functioning and vice versa, which makes interpretation of the estimates more intuitive.

**Table 2:** OLS and IV regression results: Colour Trail Tasks 1 and 2

	OLS		First-Stage IV		Reduced Form		Second-Stage IV	
	(1)	(2)	(3)	(4)	(5)	(6)	(7)	(8)
Dependent Variable	–ln(CTT1)	–ln(CTT2)	RetDur	RetDur	–ln(CTT1)	–ln(CTT2)	–ln(CTT1)	–ln(CTT2)
*MarBar*	––	–––	4.777***	4.794***	0.00481	0.0100	––	––
			(5.9)	(5.9)	(0.2)	(0.6)		
*RetDur*	–0.00193***	–0.00142**	–––	––	––	––	0.0010	0.0021
	(–3.4)	(–3.2)					(0.2)	(0.6)
*Age*	–0.0212***	–0.0167***	0.892***	0.882***	–0.0230***	–0.0181***	–0.024***	–0.020***
	(–21.2)	(–21.6)	(29.7)	(28.9)	(–26.3)	(–26.6)	(–5.1)	(–5.6)
*School*	0.0111***	0.0134***	–0.566***	–0.560***	0.0122***	0.0141***	0.013***	0.015***
	(3.8)	(5.8)	(–5.6)	(–5.5)	(4.1)	(6.2)	(3.1)	(4.9)
*NoBooks*	–0.0559***	–0.0817***	0.458	0.552	–0.0563***	–0.0817***	–0.057***	–0.083***
	(–3.4)	(–6.4)	(0.8)	(1.0)	(–3.4)	(–6.3)	(–3.4)	(–6.3)
*PoorHealth*	–0.0684*	–0.0476*	0.180	0.113	–0.0682*	–0.0472^†^	–0.068*	–0.047^†^
	(–2.2)	(–2.0)	(0.2)	(0.1)	(–2.2)	(–2.0)	(–2.2)	(–1.9)
*PoorFam*	–0.00953	–0.0172	0.657	0.744	–0.00990	–0.0173	–0.011	–0.019
	(–0.4)	(–1.0)	(0.9)	(1.0)	(–0.5)	(–1.0)	(–0.5)	(–1.1)
*MotherNotWork*	–0.00425	–0.0156	1.048^†^	1.160*	–0.00689	–0.0178	–0.0079	–0.020
	(–0.3)	(–1.2)	(1.9)	(2.0)	(–0.4)	(–1.4)	(–0.5)	(–1.5)
*FatherNotWork*	–0.0307	–0.0224	0.690	0.736	–0.0322	–0.0244	–0.033	–0.026
	(–1.0)	(–0.9)	(0.7)	(0.7)	(–1.1)	(–1.0)	(–1.1)	(–1.1)
Constant	–2.566***	–3.614***	–41.29***	–40.87***	–2.480***	–3.546***	–2.44***	–3.46***
	(–33.6)	(–61.3)	(–16.4)	(–16.1)	(–33.8)	(–62.6)	(–10.8)	(–19.9)
* R*^2^ (%)	26.2	29.4	33.2	32.7	25.9	29.1	––	––
*N*	2,519	2,481	2,519	2,481	2,519	2,481	2,519	2,481
First-Stage IV Statistics								
*F* statistics	––	––	35.14	34.97	––	––	––	––
Stock-Yogo Weak Identification Test critical values								
10 % maximal IV size	––	––	16.38	16.38	––	––	––	––
15 % maximal IV size	––	––	8.96	8.96	––	––	––	––
20 % maximal IV size	––	––	6.66	6.66	––	––	––	––
25 % maximal IV size	––	––	5.53	5.53	––	––	––	––
Hausman Test (H_0_: *RetDur* is exogenous)								
χ^2^	––	––	––	––	––	––	0.37	0.89
*p* value	––	––	––	––	––	––	.54	.34
OLS or IV?	––	––	––	––	––	––	OLS	OLS

*Notes: t* statistics are shown in parentheses. Abbreviations: CTT1 = Colour Trail Task 1; CTT2 = Colour Trail Task 2.

^†^*p* < .10; *p < .05; **p < .01; ***p < .001

**Table 3:** OLS and IV regression results: Choice reaction time and variability

	OLS		First-Stage IV		Reduced Form		Second-Stage IV	
	(1)	(2)	(3)	(4)	(5)	(6)	(7)	(8)
Dependent Variable	–ln(CRT)	–ln(CRT_VAR)	RetDur	RetDur	–ln(CRT)	–ln(CRT_VAR)	–ln(CRT)	–ln(CRT_VAR)
*MarBar*	––	––	4.716***	4.716***	–0.0145	–0.0221	––	––
			(5.8)	(5.8)	(–0.7)	(–0.5)		
*RetDur*	–0.00103*	–0.00288*	––	––	––	––	–0.0031	–0.0047
	(–2.1)	(–2.5)					(–0.7)	(–0.5)
*Age*	–0.00779***	–0.0216***	0.893***	0.893***	–0.00861***	–0.0241***	–0.0059	–0.020*
	(–9.2)	(–10.8)	(28.6)	(28.6)	(–11.6)	(–13.7)	(–1.5)	(–2.1)
*School*	0.00475^†^	0.0163**	–0.538***	–0.538***	0.00530*	0.0179**	0.0036	0.015^†^
	(1.9)	(2.8)	(–5.2)	(–5.2)	(2.2)	(3.1)	(1.1)	(2.0)
*NoBooks*	–0.0459***	–0.0881**	0.492	0.492	–0.0465***	–0.0896**	–0.045**	–0.087**
	(–3.3)	(–2.7)	(0.8)	(0.8)	(–3.3)	(–2.7)	(–3.2)	(–2.6)
*PoorHealth*	–0.00604	0.0214	–0.499	–0.499	–0.00582	0.0225	–0.0074	0.020
	(–0.2)	(0.3)	(–0.4)	(–0.4)	(–0.2)	(0.4)	(–0.3)	(0.3)
*PoorFam*	–0.0166	–0.0408	0.725	0.725	–0.0180	–0.0434	–0.016	–0.040
	(–0.9)	(–1.0)	(1.0)	(1.0)	(–1.0)	(–1.0)	(–0.9)	(–0.9)
*MotherNotWork*	–0.00453	–0.0129	1.514**	1.514**	–0.00581	–0.0171	–0.0011	–0.0099
	(–0.3)	(–0.4)	(2.6)	(2.6)	(–0.4)	(–0.5)	(–0.07)	(–0.3)
*FatherNotWork*	–0.00665	–0.0368	0.0784	0.0784	–0.00704	–0.0373	–0.0068	–0.037
	(–0.3)	(–0.6)	(0.07)	(0.07)	(–0.3)	(–0.6)	(–0.3)	(–0.6)
Constant	–5.727***	–3.400***	–42.14***	–42.14***	–5.689***	–3.283***	–5.82***	–3.48***
	(–88.8)	(–22.3)	(–16.2)	(–16.2)	(–92.1)	(–22.5)	(–30.0)	(–7.6)
*R*^2^ (%)	7.9	10.3	33.1	33.1	7.7	10.1	––	––
*N*	2,383	2,383	2,383	2,383	2,383	2,383	2,383	2,383
First-Stage IV Statistics								
*F* statistics	––	––	33.37	33.37	––	––	––	––
Stock-Yogo Weak Identification Test critical values								
10 % maximal IV size	––	––	16.38	16.38	––	––	––	––
15 % maximal IV size	––	––	8.96	8.96	––	––	––	––
20 % maximal IV size	––	––	6.66	6.66	––	––	––	––
25 % maximal IV size	––	––	5.53	5.53	––	––	––	––
Hausman Test (H_0_: *RetDur* is exogenous)								
χ^2^	––	––	––	––	––	––	0.25	0.04
*p* value	––	––	––	––	––	––	.61	.85
OLS or IV?	––	––	––	––	––	––	OLS	OLS

*Notes: t* statistics are shown in parentheses. Abbreviations: CRT = Choice Reaction Time; CRT_VAR = CRT Variability.

^†^*p* < .10; *p < .05; **p < .01; ***p < .001

Since the cognition measures are transformed into natural logarithms, the regression coefficients can be easily transformed into percentage effects. For example, *%RetDur = *[exp(β_1_) – 1].

The coefficient of *RetDur* is negative for the four cognition measures, which is consistent with the hypothesis that a longer retirement duration is associated with lower cognition. Even though these associations are statistically significant at the 5 % level or lower, the magnitude is small. An additional year of retirement corresponds to a 0.2 % reduction in CTT1, a 0.1 % reduction in CTT2, a 0.1 % reduction in CRT, and a 0.3 % reduction in CRT_VAR*.* As expected, the coefficient of *Age* is negative for all four cognition measures and is statistically significant at the 1 % level. An additional year of age is associated with a reduction of 2.1 % in CTT1, 1.7 % in CTT2, 0.8 % in CRT, and 2.1 % in CRT_VAR.

The coefficient of *School* is positive and statistically significant for all cognition measures. An additional year of schooling is associated with a 1.1 % increase in CTT1, a 1.3 % increase in CTT2, a 0.5 % increase in CRT, and a 1.6 % increase in CRT_VAR. As a group, the remaining variables should proxy well the socioeconomic conditions in the home where the respondent grew up. Strong support for the hypothesis that early-life conditions’ effects on later-life cognition is found for the variable *growing up in a household with no or few books*. The coefficient of *NoBooks* is negative and statistically significant at the 1 % level for all four cognition variables. The magnitude of this association is sizable: cognition is approximately 5.7 % lower for CTT1, 8.5 % lower for CTT2, 4.7 % lower for CRT, and 9.2 % lower for CRT_VAR growing up in a household with no or few books. It is not clear, however, whether this is a socioeconomic effect or an early reading effect. Self-reported health is also important. However, the reasons behind poor childhood health can be caused not only by socioeconomic conditions but also by factors largely independent of socioeconomic conditions (such as contagious disease).

**Fig. 6:**
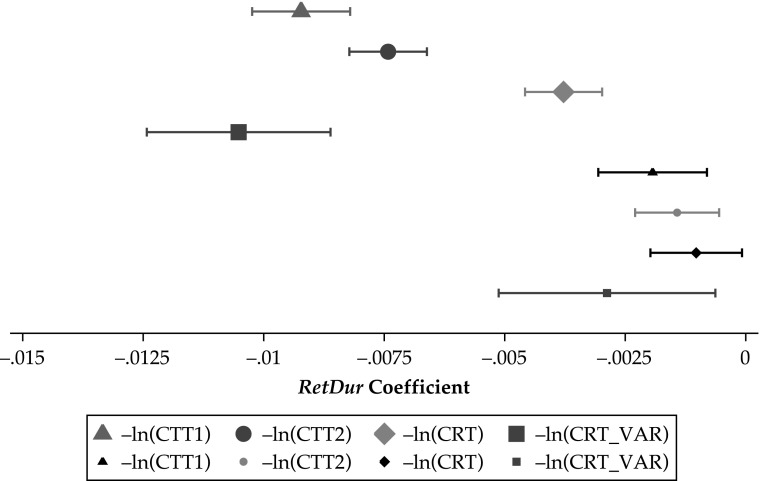
*RetDur* coefficient before and after control variables are added. *RetDur* coefficient and 95 % confidence interval are shown. Large symbols denote the *RetDur* coefficient before control variables are added. Small symbols denote the *RetDur* coefficient after control variables are added

The association of *RetDur* with CTT1, CTT2, CRT, and CRT_VAR before and after the control variables are added is visually depicted in Fig. 6. Larger symbols are used to depict the *RetDur* coefficient before the control variables are added. Smaller symbols are used to depict the *RetDur* coefficient after the control variables are added. The 95 % confidence interval of each coefficient is also shown. Figure 6 shows that after the control variables are added, the size of the *RetDur* coefficient is approximately 20 % to 25 % of the size of the initial coefficient.

The estimates of columns 1 and 2 in Tables 2 and 3 and Fig. 6 are based on the assumption that retirement duration is exogenous. The IV estimates that test for the potential endogeneity are shown in columns 3–8 in Tables 2 and 3. These columns show the first-stage IV estimates, the reduced-form estimates, and the second-stage IV estimates. As discussed in the previous section, the instrument employed is whether the woman reported having to leave a job because of the marriage bar. Columns 3 and 4 in Table 2 show the first-stage estimates for CTT1 and CTT2. There are only slight differences between the two columns because of the small differences in sample sizes. Columns 3 and 4 in Table 3 show the first-stage estimates for CRT and CRT_VAR. The two columns are identical because the sample size is the same in the two regressions.

Clearly, *MarBar* is an important predictor of *RetDur*. The coefficient of *MarBar* in all equations is positive, large in magnitude, and statistically significant at well below the 1 % level. The statistics from the first-stage equations reported at the bottom of Tables 2 and 3 confirm that the instrument is not weak (see Bound et al. 1995; Hernan and Robins 2006; Murray 2006; Staiger and Stock 1997; Stock and Yogo 2005). For example, the *F* statistics range between 33.4 and 35.1. According to Staiger and Stock’s (1997) rule of thumb, the *F* statistics should be at least 10 for the instrument not to be weak. Similarity, the Stock-Yogo tests of weak identification reject the null hypothesis that the instrument is weak given that the *F* statistics exceed the selected critical values. In short, women who had to leave work because of the marriage bar have a longer retirement duration—or more correctly, a longer current period of not working—even after we control for age and education. The requirement that the instrument is a strong predictor of the potentially endogenous variable is satisfied.

Unfortunately, we cannot directly test the requirement that there is no relationship between *MarBar* and *Cog*, apart from the indirect route via *RetDur*. However, we can obtain some information by considering the reduced-form regressions. In these regressions, CTT1, CTT2, CRT, and CRT_VAR are expressed as a function of the *MarBar* and of the other variables. These estimates are shown in columns 5 and 6 of Tables 2 and 3. *MarBar* is not statistically significant in any regression. In fact, the *t* statistics range between 0.2 and 0.7. This lack of statistical significance is encouraging and suggests that a relationship between the IV and the outcome of interest is unlikely to exist (Angrist and Krueger 2001; French and Popovici 2011).

Finally, columns 7 and 8 in Tables 2 and 3 show the estimates of the second-stage regression results. For all cognition measures, the coefficient of *RetDur* is statistically insignificant. We compare differences between the estimators of the OLS and IV by employing the Hausman test. If OLS and IV estimators are found to have a different probability limit, then there is evidence that endogeneity is present, and OLS estimators will be inconsistent. If OLS and IV estimators are found to have the same probability limit, then there is no evidence that endogeneity is present. Both estimators will be consistent, and OLS estimation is preferred. The results of the Hausman test are given at the bottom of Tables 2 and 3. For all four cognition measures, the χ^2^ values are not statistically significant, implying that the null hypothesis that retirement duration is exogenous cannot be rejected at any level of statistical significance. This leads us to conclude that the OLS estimates are preferred. More generally, there is no statistical evidence that retirement duration is endogenous. Therefore, if retirement duration and cognition are causally related, then retirement affects cognition and not the other way round.

### Robustness Checks and Model Extensions

To consider the robustness of the estimates, five sets of additional regressions are estimated (results available in Online Resource 1). The main conclusion is that the magnitude of the relationship between retirement duration and cognition remains small and statistically significant for all cognition measures.

The first set of regressions employ three alternative IVs. As explained earlier, the marriage bar was not enforced universally. It was enforced by law in the public sector and mimicked by many, but not all, private sector employers. One cannot exclude that women with certain characteristics that are not measured in the TILDA data set selected into jobs that were affected, or not affected, by the marriage bar. For example, perhaps women with an innate desire to be active in the labor force opted for jobs that would allow them to work after marriage, primarily in the private sector. If this unmeasured variable *innate desire to be active in the labor force* is also correlated with employment/retirement duration and cognition, then the IV used in the analysis is not valid.

Other unobservable characteristics that are potentially correlated with occupational choice at labor market entry and retirement duration are risk aversion and family preferences. For example, perhaps women who were more risk-averse and more family oriented opted for jobs in the public sector given that retiring at marriage was enforced by law. Similarly, perhaps women who were less risk-averse and less family-oriented opted for jobs in the private sector given that not all private sector employers enforced the marriage bar. In other terms, career prospects might have been better in the private sector. Although it is difficult to argue that traits such as risk aversion and family preferences are also correlated with cognition, one cannot exclude this might be the case.

Three IVs that are clearly independent of the occupation the woman had are constructed. The first two instruments are proxies for the number of years a woman was exposed to the marriage bar. The first instrument, *MarBarBirth*, is the time elapsed between a woman’s year of birth and 1977, which was the year when discrimination in employment on the grounds of sex or marital status was made illegal in Ireland. The second instrument, *MarBar18*, is the time elapsed between the year in which a woman turned 18 years of age and 1977. The third instrument, *PropMarBar*, is equal to the proportion of women in the TILDA sample who reported having been affected by the marriage bar by birth cohort.

The second set of regressions focus on whether the coefficient of *RetDur* is significantly different in magnitude under alternative specifications compared with what is found in the OLS baseline regressions of Tables 2 and 3. Five tests are employed. First, older women are excluded from the sample because employment rates among “older” women are very low. Second, women who performed the health assessment in their homes are excluded because they might differ from those who travelled to Trinity College Dublin to undertake the health assessment. Third, the unemployed and the sick and disabled are excluded to examine how robust the estimate of *RetDur* is to different definitions of retirement. Fourth, only those who have a retirement duration of at least one year are considered as retired. Fifth, quadratic and cubic terms in age are added to the list of explanatory factors.

The third set of regressions investigate the role of “nonwork substitution activities.” It is reasonable to hypothesize that women who retired around the time of marriage or in early adulthood substituted work activities with nonwork activities. If such activities are mentally stimulating, one would expect to find a smaller and potentially insignificant effect of retirement duration on later-life cognition for this group of women. Three tests are employed. The first test is an investigation of whether the time spent out of the labor force—associated with having children—affects later-life cognition. Perhaps the positive effect that child-rearing has on cognition outweighs the negative effect of time not working. The second test is an investigation of whether there is an association between current nonwork activities—such as volunteering—and cognition. The (untestable) assumption is that women who engage more into nonwork activities at present are more likely to have engaged in such activities in the past. The third test employs additional information on employment histories collected for women who had to leave a job because of the marriage bar.

The fourth set of regressions investigate whether the relationship between retirement and cognition can be explained by the nature of employment during one’s working life. Two tests are employed. The first test is to add an interaction term between *RetDur* and a dummy variable capturing the occupational sector of the preretirement job to the list of explanatory factors. If the cognitive stimulating nature of work is what improves cognitive function, then one can expect that the largest effects of retirement are for women in more cognitively stimulating jobs. The second test is to add an interaction term between *RetDur* and a dummy variable capturing whether employment is performed on a part-time or full-time basis. If there is a dose-response relationship between hours worked in a typical week and cognitive stimulation, then one can expect that the largest negative effects of retirement are for women in full-time jobs. However, another possibility is that women working part-time engage in equally cognitively stimulating activities when they are not in work—particularly for women who choose to retire gradually from work.

The fifth set of regressions investigates the role of cohort differences in cognitive functioning because one cannot exclude that lower duration of retirement is simply a marker for being born in a more recent birth cohort. If, *ceteris paribus*, individuals born in later generations begin adulthood with higher overall levels of performance than those born in earlier generations, then these younger participants will outperform older participants at any given time point—not because of aging-related changes but because of historical differences in, for example, nutrition or education (Tucker-Drob and Salthouse 2011). To test this hypothesis, we add an interaction term between *RetDur* and age at retirement to the list of explanatory factors.

## Conclusion

In this study, we empirically investigated the relationship between retirement duration and cognitive functioning using data for older Irish women collected in the third wave of The Irish Longitudinal Study on Ageing. Because retirement is potentially endogenous with respect to cognition, we used IV estimation. The identifying instrument was the abolition of the so-called marriage bar, which was the legal requirement that women leave paid employment upon getting married. We found a robust negative effect of retirement duration on cognition but found no support for the alternative causal direction. The finding of a negative effect of retirement duration on cognition supports the mental exercise—the “use it or lose it”—hypothesis. However, the effect of retirement duration on cognition was small in magnitude. At least three possible explanations account for our finding of a small effect.

The first explanation is that our measure of retirement duration is possibly prone to measurement error, which in turn could reduce the predictive power of the effect of retirement duration on cognition. Respondents were asked to report the date they ceased working. These self-reported responses may be subject to recall bias. In addition, there might be substantial heterogeneity in what women perceive as being work. Finally, questions on timing of labor market exit were asked slightly differently to respondents according to whether they reported to be retired, unemployed, or disabled, or looking after family. This may have created some distortion in respondents’ self-reports as to when they stopped working. TILDA data might not be of the sufficient quality needed to support the rigorous statistical analysis of the relationship between retirement and cognition.

The second explanation is that the calculation of retirement duration as “time elapsed since last stopped working” likely masks important aspects of employment histories. For example, it is reasonable to hypothesize that the estimated cognitive disadvantage associated with longer retirement duration is a lower bound of the true effect if women who retire gradually (i.e., who reduce hours of work before retirement) engage in equally stimulating cognitive activities in the newly available time before and after retirement. Similarly, it is also reasonable to hypothesize that women who have been retired for longer substituted work activities with equally cognitively stimulating nonwork activities. Information collected in TILDA on part-time versus full-time employment, current nonwork activities, and childbearing and child-rearing was used to test these hypotheses. We did not find strong evidence in favor of the substitution hypothesis. However, to investigate this with rigor would require the collection of detailed employment and life histories, which are not currently a feature of TILDA.

The third explanation is that the cognition variables employed in the analysis are based on cognitive tests that capture processing speed and mental switching, which are central to effective cognitive functioning. These tests have two important advantages. First, they are administered and scored by nurses trained specifically for this purpose. Second, they require effortful processing at the time of assessment and do not require production of previously acquired knowledge (Tucker-Drob and Salthouse 2011). However, these tests have a clear limitation. Previous investigations of aging trajectories for the processing speed factor have reported strong genetic influences on rates of cognitive decline, with little contribution from environmental factors (Finkel et al. 2005; Reynolds et al. 2005). If the validity of this finding is confirmed by future research, then it will not be surprising that the effects of retirement duration on cognition—measured by tests capturing processing speed—are small.

Another finding of our study was that the effects of education and other favorable early-life indicators on later-life cognition were positive and large in magnitude. This finding is encouraging because it suggests that educational attainment and early-life conditions may have important real-world implications for cognitive functioning in adulthood and old age (Tucker-Drob and Salthouse 2011). Whether these factors also protect from age-related cognitive decline is still the subject of debate in the literature and is beyond the scope of this study.

Our analysis was based on older Irish women. It is reasonable to hypothesize that the effects of retirement on cognition might be greater among older Irish men perhaps as a result of men being more oriented toward paid work than women or perhaps as a result of women experiencing very heterogeneous life trajectories. As a consequence, some of the analysis for women was repeated for men using TILDA data. However, we could not investigate the potential endogeneity of retirement among men because the abolition of the marriage bar is only a sensible IV for women. These estimates are not reported here but are available on request. The estimates confirm a similar relationship for men. The magnitude of the relationship is larger for men but is still small. Because it was not possible to explore the endogeneity issue for men, these estimates, albeit encouraging, are only indicative and far from conclusive.

In closing, we believe that our findings are generalizable to other high-income countries. Our analysis confirmed findings of research from other countries regarding the effect of age, education, and early-life socioeconomic conditions on later-life cognition. In this respect, Irish women appear to be no different. For the same reason, we do not believe that the key finding of a small, negative relationship between retirement duration and later-life cognition is not generalizable. However, further research based on additional data—and possibly on alternative sources of exogenous variation—is needed to further clarify the relationship between retirement and later-life cognition. Distinguishing the relative importance of the work environment and the alternative uses of time during retirement for maintaining levels of cognition in later life should be a priority.

## Electronic Supplementary Material

ESM 1 (DOCX 273 kb)
