# Supplementary material for: Effect of Retirement on Cognition: Evidence From the Irish Marriage Bar
Source: Demography. 2018 Jun 7;55(4):1317–41. doi: 10.1007/s13524-018-0682-7 (PMC6060984; doi:10.1007/s13524-018-0682-7)

**Online Resource 1**

This supplement is divided into four sections. The first section graphically illustrates the relationship between age and cognition and between retirement duration and cognition. The second section employs alternative instrumental variables (IVs) to investigate the robustness of the IV results. The third section focuses on general robustness checks. The fourth section focuses on model extension.

**1. The Relationship of Age and Retirement Duration with Cognition**

Figure S1, panels (a)-(d), plot the relationship between age and the four cognitive measures. In contrast to Figure 1, panels a-d, presented in the manuscript, the four cognitive measures CTT1, CTT2, CRT and CRT_VAR are expressed in the original metric, i.e. time taken to complete the task. The trend lines confirm that the time taken to complete the tests increases with age. This relationship is highly statistically significant (p-values < 0.01).

The figures also show the relationship between age and the standardised values (z-scores) of the cognition variables. The figures have the same right-hand scale. This transformation makes it easier to compare “how steep” the relationship with age is for each cognition variable on a similar base. Regressions were fit with standardised values of the cognition variables as the dependent variable and age as the only independent variable. The trend lines implied by these regressions are also shown in the figures. A formal statistical test suggests that there are no differences between z(CCT1) and z(CCT2) with respect to their relationship with age (p-value=0.6). Likewise, there is no difference between z(CRT) and z(CRT_VAR) (p-value=0.9). However, there is sizeable difference between z(CCT1) / z(CCT2) and z(CRT) / z(CRT_VAR), which is highly statistical significant (p-values < 0.01). The regression estimates suggest that the relationship between age and z(CCT1) / z(CCT2) is over “twice as steep” as the relationship between z(CRT) / z(CRT_VAR).

Figure S2, panels (a)-(d) plot the relationship between retirement duration and the four cognitive measures. The four cognitive measures CTT1, CTT2, CRT and CRT_VAR are expressed as time taken to complete the task. The trend lines confirm that the time taken to complete the tests increases with retirement duration. The relationships are highly statistically significant (p-value < 0.01). That is, on average, the longer an individual has been retired the lower their cognition. This is expected since, on average, people who have been retired longer are older.

In a similar manner to Figure S1, panels (a)-(d), the relationship between retirement duration and the standardised values (z-scores) of the cognition variables is also shown. Statistical tests suggest that there is no difference between z(CCT1) and z(CCT2) with respect to their relationship with retirement duration (p-value = 0.4). The same is the case for z(CRT) and z(CRT_VAR). However, the relationship is about “three times steeper” for z(CCT1) / z(CCT2) compared z(CRT) / z(CRT_VAR). It is also worth noting that the relationship between age and cognition is two to three times steeper that the relationship between retirement duration and cognition. This suggests that they are not the same and that there is likely considerable unique variation in retirement duration unrelated to age.

**2. Alternative Instrumental Variables**

The instrument used in the analysis of Tables 2 and 3 is a dummy variable, “*MarBar*”, coded “1” if a woman reported having to leave employment on getting married and “0” otherwise. As explained in the manuscript, the marriage bar was not enforced universally. It was enforced by law in the public sector and mimicked by many, but not all, private sector employers. One cannot exclude that women with certain characteristics that are not measured in the TILDA dataset selected into jobs which were affected, or not affected, by the marriage bar. For example, it could be that women with an innate desire to be active in the labor force opted for jobs that would allow them to work after marriage, primarily in the private sector. If this unmeasured variable “innate desire to be active in the labor force” is also correlated with employment/retirement duration and cognition, then the IV used in the analysis is not valid. Other unobservables that are potentially correlated with occupational choice at labor market entry and retirement duration are risk aversion and family preferences. For example it could be that women who were more risk adverse and more family oriented opted jobs in the public sector as retiring at marriage was enforced by law. Similarly, it could be that women who were less risk adverse and less family oriented opted for jobs in the private sector, as not all private sector employers enforced the marriage bar. In other terms, career prospects might have been better in the private sector. Although it is difficult to argue that traits such as risk aversion and family preferences are also correlated with cognition, one cannot exclude this might be the case.

Three IVs that are clearly independent of the occupation the woman had are constructed. The first two instruments are proxies for the number of years a woman was exposed to the marriage bar. The first instrument, *MarBarBirth*, is the time elapsed (in number of years) between a woman’s year of birth and 1977. 1977 is the year in which discrimination in employment on the grounds of sex or marital status was made illegal in Ireland. To illustrate, the instrument *MarBarBirth* is equal to 30 for a woman born in 1947, as the time elapsed between 1947 and 1977 is 30 years. The second instrument, *MarBar18*, is the time elapsed (in number of years) between the year in which a woman turned 18 years of age and 1977. Taking again a woman born in 1947 as an example, the instrument *MarBar18* is equal to 12 for such a woman, as she turned 18 in 1965. The instrument *MarBar18* is set to 0 for women born in 1959 onwards. The third instrument, *PropMarBar*, is equal to the proportion of women in the TILDA sample who reported to be affected by the marriage bar by birth cohort.

Results are presented in Tables S1 to S6. In all six tables, Columns (1) to (2) show the OLS regression estimates, which are identical to the OLS regression estimates of Tables 2 and 3 presented in the manuscript. Columns (3) to (8) show the IV estimates that test for the potential endogeneity of *RetDur*. Columns (3) to (4) show the “first stage” estimates, Columns (5) to (6) show the “reduced form” estimates and Columns (7) to (8) show the “second stage” estimates. The instrument employed in the regressions of Table S1 and S2 is *MarBarBirth*. The instrument employed in the regressions of Table S3 and S4 is *MarBar18*. The instrument employed in the regressions of Table S5 and S6 is *PropMarBar.* Tables S1, S3, and S5 show results for CTT1 and CTT2. Tables S2, S4, and S6 show results for CRT and CRT_VAR.

Focusing first on the results of Tables S1 and S2, the estimates of Columns (3) and (4) show that the instrument *MarBarBirth* is a weak predictor of *RetDur*. The t-statistics of *MarBarBirth* ranges between 2 and 2.2, and the F-statistics ranges between 4 and 4.8. According to Staiger and Stock’s (1997) rule of thumb, the F-statistics should be at least 10 for the instrument not to be weak. The F-statistics also does not exceed the critical values identified in the Stock-Yogo weak identification test (Stock and Yogo, 2005). It is not surprising that this instrument is not a strong predictor of *RetDur* as it measures the time elapsed between birth and 1977. To illustrate, a woman born in 1962 was “technically” born too late to be affected the marriage bar as she only turned 15 in 1977. Still, the variable *MarBarBirth* is equal to 15 years for this woman.  For completeness, the reduced form and second stage estimates are also shown in Tables S1 and S2 in Columns (5) to (8). These estimates, however, cannot be trusted as the estimates of the first stage regression indicate that the instrument *MarBarBirth* is a weak predictor of *RetDur*. As Murray (2006, p. 114) clearly states, “researchers need to guard against drawing misleading inferences from weak instruments”.

Turning then to the results of Tables S3 and S4, the estimates of Columns (3) and (4) show that the instrument *MarBar18* is an important predictor of *RetDur.* The coefficient of *MarBar18* in all first stage equations is positive, large in magnitude and statistically significant at well below the 1% level (t-statistics ranging between 7.6 and 7.7). The first-stage F-statistics is large. The reduced form estimates of Columns (5) and (6) show that the t-statistics of the instrument *MarBar18* is statistically significant below the 5% level only in the regression of Column (5) in Table S3. This regression expresses CTT1 as a function of *MarBar18* and of the other variables. The second stage estimates of Columns (7) and (8) show that for CTT2 the coefficient of *RetDur* is negative and statistically significant at the 10% level. For CRT and CRT_VAR, the coefficient of *RetDur* is negative and statistically insignificant. For CTT2, CRT and CRT_VAR, the OLS estimates are preferred as there is no statistical evidence that retirement duration is endogenous. For CTT1, the coefficient of *RetDur* is negative and statistically significant below the 1% level in the second stage equation. For this cognitive measure, the IV estimates are preferred as there is statistical evidence that retirement duration is endogenous. However, it is important to note that CTT1 is also the cognitive measure that is significantly associated with the instrument *MarBar18* as shown in the reduced form equation of Column (5) in Table S3.

Turning finally to the results of Tables S5 and S6, the estimates of Columns (3) and (4) in Table S5 show that the instrument *PropMarBar* is an important predictor of *RetDur.* The coefficient of *PropMarBar* in the first stage equations is positive and statistically significant at well below the 1% level (t-statistics of 3.5). The coefficient of *PropMarBar* is very large in magnitude, suggesting that on average *RetDur* is 16.6-16.8 years longer for women born in years where all women were affected by the marriage bar compared to women born in years where no women were affected by the marriage bar. The estimates of Columns (3) and (4) in Table S6 and the first stage equation statistics show that the instrument *PropMarBar* is a weaker predictor of *RetDur* for the smaller sample of women who undertook the cognitive tests CRT and CRT_VAR. In the first stage regressions of Columns (3) and (4) in Table S6, the t-statistics of the coefficient of *PropMarBar* is 2.6, resulting in a F-statistics of 6.8, which is once again below 10 (Staiger and Stock, 1997).

The second stage estimates of Columns (7) and (8) in Tables S5 and S6 show that for CTT1, CTT2 and CRT_VAR, the coefficient of *RetDur* is statistically insignificant. For these three cognition measures, the OLS estimates are preferred as there is no statistical evidence that retirement duration is endogenous. For CRT, the coefficient of *RetDur* is positive and statistically significant below the 10% level. For this cognitive measure, the IV estimates are preferred as there is statistical evidence that retirement duration is endogenous. However, it is important to note that CRT is also the cognitive measure that is significantly associated with the instrument *PropMarBar18* as shown in the reduced form equation of Column (5) in Table S6. In addition, the instrument *PropMarBar18* is weak.

**3. General Robustness Checks**

The general robustness checks focus on whether the coefficient of *RetDur* is significantly different in magnitude under alternative specifications compared to what is found in the OLS “baseline” regressions of Tables 2 and 3. Regressions are fit with restricted samples or with different/modified lists of explanatory factors. A Wald test is used to test the cross-equation restriction that the coefficient of *RetDur* from each of these regressions is not statistically different from the coefficient of *RetDur* of the baseline regression. Results are presented in Table S7 and discussed below.

Exclusion of Older Respondents

There are two tests that consider how robust the estimate of *RetDur* is to excluding “older” women from the sample, as employment rates among “older” women are very low. This is done by restricting the sample to women less than age 80, and then to women less than age 70. In both sample restrictions, the coefficient of *RetDur* remains negative and statistically significant at the 5% level or lower. The Wald test confirms that these estimates are not statistically different to the baseline estimates.

Exclusion of Home Assessment Respondents

The next test excludes those women who performed the health assessment in their homes. A priori, one might expect these women to be different from those who travelled to the health centre in Trinity College Dublin to undertake the health assessment. In this sense, the women who undertook the health assessment in the health centre may be a self-selected sample. Taking the 10% level (p=0.1) as the statistically significant threshold level, the results of Table S7 show that the coefficient of *RetDur* remains negative and statistically significant for three of the cognition measures employed when the sample is restricted in this way. It is negative but statistically insignificant for the CRT measure. The Wald test confirms that the four estimates are not statistically different to the baseline estimates.

Definition of Retirement

There are three tests that examine how robust the estimate of *RetDur* is to different definitions of retirement. The first excludes unemployed women from the sample. The second excludes sick and disabled women from the sample. The third defines as being retired those who have a retirement duration of at least one year. When unemployed women are excluded, the coefficient of *RetDur* remains negative and statistically significant at the 5% level or lower, with the Wald test confirming that the estimates are not statistically different to the baseline estimates. When the sick and disabled are excluded, the coefficient of *RetDur* remains negative but is smaller in magnitude. The Wald test confirms that this difference is statistically significant at the 5% level for CTT1 and 10% level for CTT2. However, in our view, these small estimated effects are only slightly smaller than the already very small effects estimated by OLS in the baseline model. The estimates appear to be robust to the using of a one year threshold to define retirement.

Quadratic and Cubic Terms in Age

In the baseline estimates, age is assumed to impact on cognition in a linear manner, which implies a constant “on average” percentage decline with age. There is some evidence that the relationship between age and cognition is non-linear, declining with age but at a diminishing rate. In order to explore this, regressions were estimated with quadratic and cubic terms in age added as explanatory factors. As Table S7 suggests, the hypothesis of non-linearity is supported. The table shows the point estimate of the coefficient of *RetDur* evaluated at the mean age of the sample. In all cases, this effect is negative. The Wald test indicated that this estimate is smaller than the baseline estimate at the 10% level or below in seven of the eight tests carried out. However, examination of the predicted relationship between age and cognition implied by these quadratic and cubic specifications of age suggest that the turning points are beyond the age of 90, implying a negative relationship across the age range observed in the sample.

**4. Model Extensions**

Non-work Substitution Activities

The empirical focus has been on the impact of time not working since the woman stopped working the “final time”. It is reasonable to hypothesise that women who retired around marriage or, more generally, in early adulthood may have substituted work activities with non-work activities. If such activities are mentally stimulating, one would expect to find a smaller and potentially insignificant impact of retirement duration on later-life cognition for this group of women. To investigate this with rigour would require the collection of detailed employment and life histories, which are not usually a priority in aging surveys and not currently a feature of TILDA. If detailed information on employment and life histories is not available, an alternative but less rigorous approach is to use information on current non-work activities or on major life events for which retrospective information has been collected, including childbearing and child-rearing. Three tests are employed in the analysis of this paper.

Childbearing and Child-rearing

The first test is an investigation of whether the time spent out of the labor force, associated with children, impacts on later-life cognition. It could be the case that the positive impact that childrearing has on cognition outweighs the negative impact of time not working. Four variables relating to: whether the woman has children (*HasChild*); the total number of children (*NumChild*); the age of youngest child (*AgeYoungChild*) and age of oldest child (*AgeOldChild*) are added to the list of explanatory factors.

Results for CTT1 and CTT2 are presented in Table S8. The coefficient of *RetDur* is negative and statistically significant at the 1% level or lower in all eight regressions. The Wald test for comparison with baseline *RetDur* is reported at the bottom of the table. This test suggests that the *RetDur* estimated effects are larger than the baseline estimated effects at the 5% level or lower. It is interesting to note that the coefficients of the variables capturing whether the woman has children or the number of children the woman has, are positive and statistically significant at the 1% level or lower in four of the eight regressions. Results for CRT and CRT_VAR are presented in Table S9. The coefficient of *RetDur* is negative and statistically significant at the 5% level or lower in all eight regressions. The Wald test suggests that the *RetDur* estimated effects are generally not different to the baseline estimates. The coefficients of the fertility variables are mostly statistically insignificant.

Current Non-work Activities

The second test is an investigation of whether there is an association between current non-work activities and cognition. The (untestable) assumption is that women who engage more into non-work activities at present or have engaged more in such activities in the recent past are also more likely to have engaged in such activities across the adult life-span. At each wave of data collection, respondents are asked about their participation in a number of social activities, including volunteering. Information is collected through the question “How often, if at all, do you do voluntary activity?” This information is used to investigate whether women who retired around marriage or more generally in early adulthood are more likely to participate frequently in volunteering activities than women who did not retire in early adulthood.

Three different cut-off points are chosen to differentiate between ‘early’ and ‘non-early’ adulthood: age 30; age 35 and age 40. There are a total of 1,552 women in the sample who are retired at Wave 3 interview, have ever been married and answered the volunteering questions at Wave 1. Around 16% of these retired before age 30, 21% retired before age 35 and 23% retired before age 40. Results are presented in Table S10. Around 21.6% of women who retired before age 30 were volunteering once a week or more at Wave 1 data collection. This compares to 19.4% of women who retired at or after turning 30. The difference between the two groups is not statistically significant. Similar patterns are found for women who retired before or after age 35 and age 40. Based on these results, it is difficult to assess the extent to which women who retired ‘early’ substituted work with non-work activities.

Employment Histories for Women Affected by the Marriage Bar

The third test employs additional information on employment histories which is collected for women who had to leave a job because of the marriage bar. This group of women are asked whether they ever returned to full-time employment, and in which year they returned. Further investigation of the TILDA data shows that around 43% (N=138) of women who left a job because of the marriage bar subsequently went back to full-time employment. They spent, on average, 12.3 years (SD = 9.9) out of the labor force before moving back into employment. A total of 39 women spent 20 years or more outside the labor force before returning to work. This information is used to investigate whether the effect of *RetDur* remains negative and statistically significant when for these 39 women retirement duration is calculated as time elapsed since marriage, and not as time elapsed since last job ended. *RetDur* is calculated as time elapsed since last job ended for the other 2,480 women in the sample. Results are presented in Table S11. The coefficient of *RetDur* is negative and statistically significant at the 1% level or lower for the four cognition measures. The Wald test indicates that the *RetDur* estimated effects are not different to the baseline estimates.

Nature of Employment

*Manual and Non-Manual Employment*

It is reasonable to hypothesize that the relationship between retirement and cognition can be explained by the nature of employment during one’s working life. If it is the cognitive stimulating nature of work that improves cognitive function, then one can expect that the largest effects of retirement are for women in cognitively stimulating jobs. To test this hypothesis, an interaction term between *RetDur* and a dummy variable capturing the occupational sector of the pre-retirement job, *NonManual,* is added to the list of explanatory factors.

In Ireland, the Central Statistics Office classify occupations into six social class groups: 1) Professional; 2) Managerial/Technical; 3) Non-manual; 4) Skilled Manual; 5) Semi-skilled; 6) Unskilled. We use different combinations of these six categories to create the *NonManual* variable needed for the test as it is reasonable to assume that the cognitive stimulating nature of work varies across the social class groups. The first definition is women are classified as being in non-manual employment (*NonManual*=1) if in social class groups “1”, “2” or “3” and in manual employment (*NonManual*=0) if in social class groups is “4”, “5” or “6”. The second is where non-manual employment is defined as social class groups “1” (Professional) or “2” (Managerial/Technical). The third is where non-manual employment is defined as social class group “1”. Varying the definition for what constitutes non-manual and manual employment is a way of testing robustness yet keeping the statistical test simple.

Table S12 reports the regression estimates that include an interaction between retirement duration and non-manual employment (*NonManual*RetDur*). If the rate of cognitive decline is different across individuals who are working in non-manual versus manual occupations, you would expect the coefficient of the interaction not to be zero. There is little evidence in support of this hypothesis. In the regressions summarised in Columns (5) to (12), the interaction term is not statistically significant at conventional threshold levels. However in three of the four regressions summarised in Columns (1) to (4), the interaction is positive and statistical significant. For the CTT1 and CTT2 cognition variables, the interaction is highly statistically significant. However, despite this statistical significance, the difference between non-manual and manual employment is small in magnitude. More specifically, evaluated at sample means, the estimates in Columns (1) to (4) imply that the cognitive advantage associated with non-manual employment relative to manual employment does not exceed 1.1 per cent. Based on the estimates in Table S12, there is little evidence of substantially significant differences in the impact of retirement duration on cognition associated with non-manual versus manual employment.

*Part-time and Full-time Employment*

It is also reasonable to hypothesize that the relationship between retirement and cognition can be explained by another important characteristic of employment during one’s working life: part-time versus full-time employment. If there is a dose-response relationship between “hours worked in a typical week” and cognitive stimulation, then one can expect that the largest negative effects of retirement are for women in full-time jobs. However, it could also be that women working part-time engage into equally cognitively stimulating activities when they are not in work. This could be particularly the case for women who choose to retire from work “gradually”. Under this scenario, one can expect the negative effect of retirement on cognition for women leaving part-time jobs to be the same or even smaller than the effect of retirement for women leaving full-time jobs. In order to test this possibility of “gradual retirement” with rigour would require the collection of detailed employment and life histories, which are not available in TILDA.

In TILDA, the information collected on “hours worked in a typical week” is limited. Questions on hours worked per week are not asked to all respondents. They are asked to employees and self-employed individuals in the farming industry in relation to their current job and to retirees in relation to their last job. They are not asked to respondents who report to be unemployed, permanently sick or disabled, in education and training, looking after family and in self-employment in an industry other than farming. Hours worked per week in current or most recent job are computed for respondents for whom information is available. In the regressions of Table S13, an interaction term between *RetDur* and a dummy variable capturing part-time employment, *PartTime*, is added to the list of explanatory factors to test whether the relationship between retirement duration and cognition can be explained by part-time and full-time employment.

We use different three different combinations of hours worked per week to create the *PartTime* variable needed for the test. The first definition is women are classified as being in part-time employment (*PartTime*=1) if average hours worked per week range between 1 and 20 and in full-time employment (*PartTime*=0) if they range between 21 and 40+. The second is where part-time employment is defined as 1 to 25 hours per week. The third is where part-time employment is defined as 1 to 30. Varying the definition for what constitutes part-time and full-time employment is a way of testing robustness yet keeping the statistical test simple.

Table S13 reports the regression estimates that include an interaction between retirement duration and part-time employment (*PartTime*RetDur*). If the rate of cognitive decline is different across individuals who are working in part-time versus full-time occupations, you would expect the coefficient of the interaction not to be zero. There is little evidence in support of this hypothesis. The interaction is positive and statistical significant only in Columns (3) and (7). However, despite this statistical significance, the difference between part-time and full-time employment is small in magnitude. More specifically, evaluated at sample means, the estimates in Columns (3) and (7) imply that the cognitive advantage associated with part-time employment relative to full-time employment does not exceed 0.4 per cent. Based on the estimates in Table S13, there is little evidence of substantially significant differences in the impact of retirement duration on cognition associated with part-time versus full-time employment.

Age of Retirement and Retirement Duration

One potential problem is that there may be cohort differences in cognitive functioning. If, *ceteris paribus*, individuals born in later generations begin adulthood with higher overall levels of performance than those born in earlier generations then these younger participants will outperform older participants at any given time point, not because of aging-related changes, but because of historical differences in, for example, nutrition or education (Tucker-Drob and Salthouse 2011). In the literature, this is usually referred to as the ‘Flynn effect’ (Flynn 1987). In relation to the analysis of this paper, one cannot exclude that lower duration of retirement is simply a marker for being born in a more recent birth cohort. To investigate this with rigour would require replicating the analysis using data from a sample with similar characteristics and composition but born later, typically a refreshment sample. If data from a refreshment sample are not available, an alternative but clearly less rigorous approach is to add an interaction term between *RetDur* and age at retirement, *AgeRet,* to the list of explanatory factors. It is important to note that as *Age* = *AgeRet + RetDur*, *Age* has to be dropped from the list of explanatory factors if *AgeRet* and *RetDur* are controlled for to avoid perfect multicollinearity.

The regression estimates based on this specification are summarised in Table S14. In these regressions, *RetDur and AgeRet* have negative sign and are statistically significant. The interaction *RetDur***AgeRet* has negative sign and is statistically significant at the 5% or lower in two of the four regressions. This suggests that, on average, women with a longer retirement duration and younger age of retirement have lower cognition than expected given their retirement duration and age at retirement. This finding is consistent with cohort effects but the interaction is very small in magnitude, indicating that any such cohort effects are small relative to the effects of retirement duration and age at retirement (and indirectly age) on cognition.

**Table S1** OLS and IV regression results, Colour Trail Task 1 and 2. IV defined as time elapsed between birth and 1977 (*MarBarBirth*)

|  | OLS | | First Stage IV | | Reduced Form | | Second Stage IV | |
| --- | --- | --- | --- | --- | --- | --- | --- | --- |
|  | (1) | (2) | (3) | (4) | (5) | (6) | (7) | (8) |
| Dependent Variable | -ln(CTT1) | -ln(CTT2) | RetDur | RetDur | -ln(CTT1) | -ln(CTT2) | -ln(CTT1) | -ln(CTT2) |
| *MarBarBirth* | –– | –– | 1.156* | 1.120* | 0.00791 | 0.00570 | –– | –– |
|  | –– | –– | (2.2) | (2.1) | (0.5) | (0.5) | –– | –– |
| *RetDur* | -0.00193^***^ | -0.00142^**^ | –– | –– | –– | –– | 0.00685 | 0.00509 |
|  | (-3.4) | (-3.2) | –– | –– | –– | –– | (0.5) | (0.5) |
| *Age* | -0.0212^***^ | -0.0167^***^ | -0.220 | -0.192 | -0.0309* | -0.0237* | -0.0294* | -0.0227* |
|  | (-21.2) | (-21.6) | (-0.4) | (-0.4) | (-2.0) | (-2.0) | (-2.3) | (-2.2) |
| *School* | 0.0111^***^ | 0.0134^***^ | -0.566*** | -0.563*** | 0.0123*** | 0.0142*** | 0.0161† | 0.0170** |
|  | (3.8) | (5.8) | (-5.5) | (-5.5) | (4.1) | (6.2) | (1.9) | (2.6) |
| *NoBooks* | -0.0559^***^ | -0.0817^***^ | 0.433 | 0.509 | -0.0566*** | -0.0823*** | -0.0595** | -0.0849*** |
|  | (-3.4) | (-6.4) | (0.7) | (0.9) | (-3.4) | (-6.4) | (-3.3) | (-5.9) |
| *PoorHealth* | -0.0684^*^ | -0.0476^*^ | -0.0199 | -0.0777 | -0.0688* | -0.0478* | -0.0686* | -0.0474† |
|  | (-2.2) | (-2.0) | (-0.02) | (-0.07) | (-2.2) | (-2.0) | (-2.1) | (-1.9) |
| *PoorFam* | -0.00953 | -0.0172 | 0.346 | 0.434 | -0.0106 | -0.0181 | -0.0130 | -0.0203 |
|  | (-0.4) | (-1.0) | (0.5) | (0.6) | (-0.5) | (-1.1) | (-0.6) | (-1.1) |
| *MotherNotWork* | -0.00425 | -0.0156 | 1.141* | 1.245* | -0.00697 | -0.0177 | -0.0148 | -0.0240 |
|  | (-0.3) | (-1.2) | (2.0) | (2.2) | (-0.4) | (-1.4) | (-0.6) | (-1.2) |
| *FatherNotWork* | -0.0307 | -0.0224 | 0.428 | 0.473 | -0.0323 | -0.0236 | -0.0352 | -0.0260 |
|  | (-1.0) | (-0.9) | (0.4) | (0.4) | (-1.1) | (-1.0) | (-1.1) | (-1.0) |
| *Constant* | -2.566^***^ | -3.614^***^ | 0.0863 | -0.905 | -2.182*** | -3.336*** | -2.182*** | -3.332*** |
|  | (-33.6) | (-61.3) | (0.004) | (-0.04) | (-3.8) | (-7.5) | (-3.6) | (-7.0) |
|  |  |  |  |  |  |  |  |  |
| *R^2^ (%)* | 26.2 | 29.4 | 32.2 | 31.9 | 25.9 | 29.1 | –– | –– |
| *N* | 2,519 | 2,481 | 2,519 | 2,481 | 2,519 | 2,481 | 2,519 | 2,481 |
| First Stage IV Statistics | | | | | | | | |
| *F-statistics* | –– | –– | 4.83 | 4.50 | –– | –– | –– | –– |
| *Stock-Yogo Weak Identification Test Critical Values:* | | | | | | | | |
| *10% maximal IV size* | –– | –– | 16.38 | 16.38 | –– | –– | –– | –– |
| *15% maximal IV size* | –– | –– | 8.96 | 8.96 | –– | –– | –– | –– |
| *20% maximal IV size* | –– | –– | 6.66 | 6.66 | –– | –– | –– | –– |
| *25% maximal IV size* | –– | –– | 5.53 | 5.53 | –– | –– | –– | –– |
| Hausman test (H_0_: RetDur is exogenous) | | | | | | | | |
| *χ2* | –– | –– | –– | –– | –– | –– | 0.45 | 0.39 |
| *P-value* | –– | –– | –– | –– | –– | –– | 0.50 | 0.53 |
| *OLS or IV?* | –– | –– | –– | –– | –– | –– | OLS | OLS |
| *Notes*: t statistics in parentheses; † p<.10, * p<.05, ** p<.01, *** p<.001. Abbreviations: CTT1: Colour Trail Task 1. CTT2: Colour Trail Task 2 | | | | | | | | |

**Table S2** OLS and IV regression results, Choice Reaction Time and Variability. IV defined as time elapsed between birth and 1977 (*MarBarBirth*)

|  | OLS | | First Stage IV | | Reduced Form | | Second Stage IV | |
| --- | --- | --- | --- | --- | --- | --- | --- | --- |
|  | (1) | (2) | (3) | (4) | (5) | (6) | (7) | (8) |
| Dependent Variable | -ln(CRT) | -ln(CRT_VAR) | RetDur | RetDur | -ln(CRT) | -ln(CRT_VAR) | -ln(CRT) | -ln(CRT_VAR) |
| *MarBarBirth* | –– | –– | 1.094* | 1.094* | 0.0229† | 0.0633* | –– | –– |
|  | –– | –– | (2.0) | (2.0) | (1.8) | (2.1) | –– | –– |
| *RetDur* | -0.00103^*^ | -0.00288^*^ | –– | –– | –– | –– | 0.0210 | 0.0579 |
|  | (-2.1) | (-2.5) | –– | –– | –– | –– | (1.3) | (1.4) |
| *Age* | -0.00779^***^ | -0.0216^***^ | -0.154 | -0.154 | -0.0318* | -0.0878** | -0.0285† | -0.0788* |
|  | (-9.2) | (-10.8) | (-0.3) | (-0.3) | (-2.5) | (-2.9) | (-1.9) | (-2.1) |
| *School* | 0.00475^†^ | 0.0163^**^ | -0.538*** | -0.538*** | 0.00537* | 0.0180** | 0.0167† | 0.0492* |
|  | (1.9) | (2.8) | (-5.2) | (-5.2) | (2.2) | (3.1) | (1.8) | (2.1) |
| *NoBooks* | -0.0459^***^ | -0.0881^**^ | 0.463 | 0.463 | -0.0461*** | -0.0889** | -0.0559** | -0.116* |
|  | (-3.3) | (-2.7) | (0.8) | (0.8) | (-3.3) | (-2.7) | (-2.8) | (-2.2) |
| *PoorHealth* | -0.00604 | 0.0214 | -0.675 | -0.675 | -0.00605 | 0.0214 | 0.00810 | 0.0604 |
|  | (-0.2) | (0.3) | (-0.6) | (-0.6) | (-0.2) | (0.3) | (0.2) | (0.6) |
| *PoorFam* | -0.0166 | -0.0408 | 0.372 | 0.372 | -0.0181 | -0.0449 | -0.0259 | -0.0664 |
|  | (-0.9) | (-1.0) | (0.5) | (0.5) | (-1.0) | (-1.1) | (-1.0) | (-1.0) |
| *MotherNotWork* | -0.00453 | -0.0129 | 1.603** | 1.603** | -0.00713 | -0.0202 | -0.0408 | -0.113 |
|  | (-0.3) | (-0.4) | (2.8) | (2.8) | (-0.5) | (-0.6) | (-1.3) | (-1.4) |
| *FatherNotWork* | -0.00665 | -0.0368 | -0.146 | -0.146 | -0.00815 | -0.0409 | -0.00509 | -0.0325 |
|  | (-0.3) | (-0.6) | (-0.1) | (-0.1) | (-0.3) | (-0.7) | (-0.1) | (-0.4) |
| *Constant* | -5.727^***^ | -3.400^***^ | -3.271 | -3.271 | -4.811*** | -0.870 | -4.742*** | -0.680 |
|  | (-88.8) | (-22.3) | (-0.2) | (-0.2) | (-9.9) | (-0.8) | (-6.7) | (-0.4) |
|  |  |  |  |  |  |  |  |  |
| *R^2^ (%)* | 7.9 | 10.3 | 32.3 | 32.3 | 7.8 | 10.2 | –– | –– |
| *N* | 2,383 | 2,383 | 2,383 | 2,383 | 2,383 | 2,383 | 2,383 | 2,383 |
| First Stage IV Statistics | | | | | | | | |
| *F-statistics* | –– | –– | 4.16 | 4.16 | –– | –– | –– | –– |
| *Stock-Yogo Weak Identification Test Critical Values:* | | | | | | | | |
| *10% maximal IV size* | –– | –– | 16.38 | 16.38 | –– | –– | –– | –– |
| *15% maximal IV size* | –– | –– | 8.96 | 8.96 | –– | –– | –– | –– |
| *20% maximal IV size* | –– | –– | 6.66 | 6.66 | –– | –– | –– | –– |
| *25% maximal IV size* | –– | –– | 5.53 | 5.53 | –– | –– | –– | –– |
| Hausman test (H_0_: RetDur is exogenous) | | | | | | | | |
| *χ2* | –– | –– | –– | –– | –– | –– | 3.6 | 4.9 |
| *P-value* | –– | –– | –– | –– | –– | –– | 0.06 | 0.03 |
| *OLS or IV?* | –– | –– | –– | –– | –– | –– | OLS | IV |
| *Notes*: t statistics in parentheses; † p<.10, * p<.05, ** p<.01, *** p<.001. Abbreviations: CRT: Choice Reaction Time. CRT_VAR: CRT Variability | | | | | | | | |

**Table S3** OLS and IV regression results, Colour Trail Task 1 and 2. IV defined as time elapsed between year of 18^th^ birthday and 1977 (*MarBar18*)

|  | OLS | | First Stage IV | | Reduced Form | | Second Stage IV | |
| --- | --- | --- | --- | --- | --- | --- | --- | --- |
|  | (1) | (2) | (3) | (4) | (5) | (6) | (7) | (8) |
| Dependent Variable | -ln(CTT1) | -ln(CTT2) | RetDur | RetDur | -ln(CTT1) | -ln(CTT2) | -ln(CTT1) | -ln(CTT2) |
| *MarBar18* | –– | –– | 1.486*** | 1.475*** | -0.0164** | -0.00736† | –– | –– |
|  | –– | –– | (7.6) | (7.6) | (-2.9) | (-1.7) | –– | –– |
| *RetDur* | -0.00193^***^ | -0.00142^**^ | –– | –– | –– | –– | -0.0111** | -0.00499† |
|  | (-3.4) | (-3.2) | –– | –– | –– | –– | (-2.8) | (-1.7) |
| *Age* | -0.0212^***^ | -0.0167^***^ | -0.459* | -0.455* | -0.00750 | -0.0111** | -0.0126** | -0.0134*** |
|  | (-21.2) | (-21.6) | (-2.5) | (-2.5) | (-1.4) | (-2.7) | (-3.2) | (-4.7) |
| *School* | 0.0111^***^ | 0.0134^***^ | -0.599*** | -0.596*** | 0.0126*** | 0.0143*** | 0.00596 | 0.0113*** |
|  | (3.8) | (5.8) | (-5.9) | (-5.9) | (4.3) | (6.3) | (1.6) | (4.0) |
| *NoBooks* | -0.0559^***^ | -0.0817^***^ | 0.450 | 0.517 | -0.0570*** | -0.0825*** | -0.0521** | -0.0799*** |
|  | (-3.4) | (-6.4) | (0.8) | (0.9) | (-3.4) | (-6.4) | (-3.0) | (-6.1) |
| *PoorHealth* | -0.0684^*^ | -0.0476^*^ | 0.139 | 0.0744 | -0.0698* | -0.0481* | -0.0683* | -0.0478† |
|  | (-2.2) | (-2.0) | (0.1) | (0.07) | (-2.2) | (-2.0) | (-2.1) | (-2.0) |
| *PoorFam* | -0.00953 | -0.0172 | 0.343 | 0.428 | -0.00977 | -0.0176 | -0.00597 | -0.0155 |
|  | (-0.4) | (-1.0) | (0.5) | (0.6) | (-0.5) | (-1.1) | (-0.3) | (-0.9) |
| *MotherNotWork* | -0.00425 | -0.0156 | 1.173* | 1.270* | -0.00626 | -0.0173 | 0.00671 | -0.0109 |
|  | (-0.3) | (-1.2) | (2.1) | (2.3) | (-0.4) | (-1.4) | (0.4) | (-0.8) |
| *FatherNotWork* | -0.0307 | -0.0224 | 0.467 | 0.510 | -0.0312 | -0.0230 | -0.0260 | -0.0204 |
|  | (-1.0) | (-0.9) | (0.4) | (0.5) | (-1.0) | (-1.0) | (-0.8) | (-0.9) |
| *Constant* | -2.566^***^ | -3.614^***^ | 33.22** | 32.90** | -3.333*** | -3.933*** | -2.966*** | -3.769*** |
|  | (-33.6) | (-61.3) | (3.2) | (3.2) | (-11.0) | (-16.9) | (-15.5) | (-26.6) |
|  |  |  |  |  |  |  |  |  |
| *R^2^ (%)* | 26.2 | 29.4 | 33.8 | 33.3 | 26.2 | 29.2 | –– | –– |
| *N* | 2,519 | 2,481 | 2,519 | 2,481 | 2,519 | 2,481 | 2,519 | 2,481 |
| First Stage IV Statistics | | | | | | | | |
| *F-statistics* | –– | –– | 57.72 | 57.06 | –– | –– | –– | –– |
| *Stock-Yogo Weak Identification Test Critical Values:* | | | | | | | | |
| *10% maximal IV size* | –– | –– | 16.38 | 16.38 | –– | –– | –– | –– |
| *15% maximal IV size* | –– | –– | 8.96 | 8.96 | –– | –– | –– | –– |
| *20% maximal IV size* | –– | –– | 6.66 | 6.66 | –– | –– | –– | –– |
| *25% maximal IV size* | –– | –– | 5.53 | 5.53 | –– | –– | –– | –– |
| Hausman test (H_0_: RetDur is exogenous) | | | | | | | | |
| *χ2* | –– | –– | –– | –– | –– | –– | 5.8 | 1.49 |
| *P-value* | –– | –– | –– | –– | –– | –– | 0.02 | 0.22 |
| *OLS or IV?* | –– | –– | –– | –– | –– | –– | IV | OLS |
| *Notes*: t statistics in parentheses; † p<.10, * p<.05, ** p<.01, *** p<.001. Abbreviations: CTT1: Colour Trail Task 1. CTT2: Colour Trail Task 2 | | | | | | | | |

**Table S4** OLS and IV regression results, Choice Reaction Time and Variability. IV defined as time elapsed between year of 18^th^ birthday and 1977 (*MarBar18*)

|  | OLS | | First Stage IV | | Reduced Form | | Second Stage IV | |
| --- | --- | --- | --- | --- | --- | --- | --- | --- |
|  | (1) | (2) | (3) | (4) | (5) | (6) | (7) | (8) |
| Dependent Variable | -ln(CRT) | -ln(CRT_VAR) | RetDur | RetDur | -ln(CRT) | -ln(CRT_VAR) | -ln(CRT) | -ln(CRT_VAR) |
| *MarBar18* | –– | –– | 1.517*** | 1.517*** | -0.00175 | -0.00450 | –– | –– |
|  | –– | –– | (7.7) | (7.7) | (-0.4) | (-0.4) | –– | –– |
| *RetDur* | -0.00103^*^ | -0.00288^*^ | –– | –– | –– | –– | -0.00116 | -0.00296 |
|  | (-2.1) | (-2.5) | –– | –– | –– | –– | (-0.4) | (-0.4) |
| *Age* | -0.00779^***^ | -0.0216^***^ | -0.481* | -0.481* | -0.00712 | -0.0201† | -0.00768* | -0.0215** |
|  | (-9.2) | (-10.8) | (-2.6) | (-2.6) | (-1.6) | (-1.9) | (-2.6) | (-3.0) |
| *School* | 0.00475^†^ | 0.0163^**^ | -0.570*** | -0.570*** | 0.00534* | 0.0180** | 0.00468 | 0.0163* |
|  | (1.9) | (2.8) | (-5.5) | (-5.5) | (2.2) | (3.1) | (1.6) | (2.3) |
| *NoBooks* | -0.0459^***^ | -0.0881^**^ | 0.492 | 0.492 | -0.0464*** | -0.0896** | -0.0458*** | -0.0881** |
|  | (-3.3) | (-2.7) | (0.8) | (0.8) | (-3.3) | (-2.7) | (-3.3) | (-2.7) |
| *PoorHealth* | -0.00604 | 0.0214 | -0.499 | -0.499 | -0.00555 | 0.0228 | -0.00613 | 0.0213 |
|  | (-0.2) | (0.3) | (-0.5) | (-0.5) | (-0.2) | (0.4) | (-0.2) | (0.3) |
| *PoorFam* | -0.0166 | -0.0408 | 0.403 | 0.403 | -0.0170 | -0.0419 | -0.0165 | -0.0408 |
|  | (-0.9) | (-1.0) | (0.5) | (0.5) | (-0.9) | (-1.0) | (-0.9) | (-1.0) |
| *MotherNotWork* | -0.00453 | -0.0129 | 1.604** | 1.604** | -0.00617 | -0.0176 | -0.00432 | -0.0128 |
|  | (-0.3) | (-0.4) | (2.8) | (2.8) | (-0.5) | (-0.5) | (-0.3) | (-0.4) |
| *FatherNotWork* | -0.00665 | -0.0368 | -0.129 | -0.129 | -0.00651 | -0.0364 | -0.00666 | -0.0368 |
|  | (-0.3) | (-0.6) | (-0.1) | (-0.1) | (-0.3) | (-0.6) | (-0.3) | (-0.6) |
| *Constant* | -5.727^***^ | -3.400^***^ | 33.55** | 33.55** | -5.772*** | -3.503*** | -5.733*** | -3.403*** |
|  | (-88.8) | (-22.3) | (3.2) | (3.2) | (-23.0) | (-5.9) | (-37.9) | (-9.5) |
|  |  |  |  |  |  |  |  |  |
| *R^2^ (%)* | 7.9 | 10.3 | 33.8 | 33.8 | 7.7 | 10.1 | –– | –– |
| *N* | 2,383 | 2,383 | 2,383 | 2,383 | 2,383 | 2,383 | 2,383 | 2,383 |
| First Stage IV Statistics | | | | | | | | |
| *F-statistics* | –– | –– | 59.24 | 59.24 | –– | –– | –– | –– |
| *Stock-Yogo Weak Identification Test Critical Values:* | | | | | | | | |
| *10% maximal IV size* | –– | –– | 16.38 | 16.38 | –– | –– | –– | –– |
| *15% maximal IV size* | –– | –– | 8.96 | 8.96 | –– | –– | –– | –– |
| *20% maximal IV size* | –– | –– | 6.66 | 6.66 | –– | –– | –– | –– |
| *25% maximal IV size* | –– | –– | 5.53 | 5.53 | –– | –– | –– | –– |
| Hausman test (H_0_: RetDur is exogenous) | | | | | | | | |
| *χ2* | –– | –– | –– | –– | –– | –– | 0.00 | 0.00 |
| *P-value* | –– | –– | –– | –– | –– | –– | 0.97 | 0.99 |
| *OLS or IV?* | –– | –– | –– | –– | –– | –– | OLS | OLS |
| *Notes*: t statistics in parentheses; † p<.10, * p<.05, ** p<.01, *** p<.001. Abbreviations: CRT: Choice Reaction Time. CRT_VAR: CRT Variability | | | | | | | | |

**Table S5** OLS and IV regression results, Colour Trail Task 1 and 2. IV defined as proportion of women affected by Marriage Bar by birth cohort (*PropMarBar*)

|  | OLS | | First Stage IV | | Reduced Form | | Second Stage IV | |
| --- | --- | --- | --- | --- | --- | --- | --- | --- |
|  | (1) | (2) | (3) | (4) | (5) | (6) | (7) | (8) |
| Dependent Variable | -ln(CTT1) | -ln(CTT2) | RetDur | RetDur | -ln(CTT1) | -ln(CTT2) | -ln(CTT1) | -ln(CTT2) |
| *PropMarBar* | –– | –– | 16.56*** | 16.78*** | -0.167 | 0.0125 | –– | –– |
|  | –– | –– | (3.5) | (3.5) | (-1.2) | (0.1) | –– | –– |
| *RetDur* | -0.00193^***^ | -0.00142^**^ | –– | –– | –– | –– | -0.0101 | 0.000745 |
|  | (-3.4) | (-3.2) | –– | –– | –– | –– | (-1.2) | (0.1) |
| *Age* | -0.0212^***^ | -0.0167^***^ | 0.775*** | 0.762*** | -0.0213*** | -0.0181*** | -0.0135^†^ | -0.0187** |
|  | (-21.2) | (-21.6) | (14.1) | (13.4) | (-13.4) | (-14.4) | (-1.7) | (-3.1) |
| *School* | 0.0111^***^ | 0.0134^***^ | -0.567*** | -0.568*** | 0.0122*** | 0.0142*** | 0.00652 | 0.0146*** |
|  | (3.8) | (5.8) | (-5.6) | (-5.5) | (4.1) | (6.2) | (1.1) | (3.4) |
| *NoBooks* | -0.0559^***^ | -0.0817^***^ | 0.438 | 0.514 | -0.0569*** | -0.0823*** | -0.0525** | -0.0827*** |
|  | (-3.4) | (-6.4) | (0.8) | (0.9) | (-3.4) | (-6.4) | (-3.0) | (-6.2) |
| *PoorHealth* | -0.0684^*^ | -0.0476^*^ | 0.0897 | 0.0345 | -0.0692* | -0.0475* | -0.0683* | -0.0475* |
|  | (-2.2) | (-2.0) | (0.1) | (0.0) | (-2.2) | (-2.0) | (-2.1) | (-2.0) |
| *PoorFam* | -0.00953 | -0.0172 | 0.289 | 0.360 | -0.00927 | -0.0179 | -0.00636 | -0.0182 |
|  | (-0.4) | (-1.0) | (0.4) | (0.5) | (-0.4) | (-1.1) | (-0.3) | (-1.1) |
| *MotherNotWork* | -0.00425 | -0.0156 | 1.146* | 1.245* | -0.00602 | -0.0175 | 0.00552 | -0.0184 |
|  | (-0.3) | (-1.2) | (2.0) | (2.2) | (-0.4) | (-1.4) | (0.3) | (-1.2) |
| *FatherNotWork* | -0.0307 | -0.0224 | 0.583 | 0.613 | -0.0324 | -0.0231 | -0.0265 | -0.0236 |
|  | (-1.0) | (-0.9) | (0.5) | (0.6) | (-1.1) | (-1.0) | (-0.8) | (-1.0) |
| *Constant* | -2.566^***^ | -3.614^***^ | -35.06*** | -34.38*** | -2.569*** | -3.546*** | -2.922*** | -3.520*** |
|  | (-33.6) | (-61.3) | (-9.9) | (-9.5) | (-25.2) | (-44.1) | (-7.7) | (-12.4) |
|  |  |  |  |  |  |  |  |  |
| *R^2^ (%)* | 26.2 | 29.4 | 32.6 | 32.1 | 26.0 | 29.1 | –– | –– |
| *N* | 2,519 | 2,481 | 2,519 | 2,481 | 2,519 | 2,481 | 2,519 | 2,481 |
| First Stage IV Statistics | | | | | | | | |
| *F-statistics* | –– | –– | 12.25 | 11.96 | –– | –– | –– | –– |
| *Stock-Yogo Weak Identification Test Critical Values:* | | | | | | | | |
| *10% maximal IV size* | –– | –– | 16.38 | 16.38 | –– | –– | –– | –– |
| *15% maximal IV size* | –– | –– | 8.96 | 8.96 | –– | –– | –– | –– |
| *20% maximal IV size* | –– | –– | 6.66 | 6.66 | –– | –– | –– | –– |
| *25% maximal IV size* | –– | –– | 5.53 | 5.53 | –– | –– | –– | –– |
| Hausman test (H_0_: RetDur is exogenous) | | | | | | | | |
| *χ2* | –– | –– | –– | –– | –– | –– | 0.98 | 0.11 |
| *P-value* | –– | –– | –– | –– | –– | –– | 0.32 | 0.73 |
| *OLS or IV?* | –– | –– | –– | –– | –– | –– | OLS | OLS |
| *Notes*: t statistics in parentheses; † p<.10, * p<.05, ** p<.01, *** p<.001. Abbreviations: CTT1: Colour Trail Task 1. CTT2: Colour Trail Task 2 | | | | | | | | |

**Table S6** OLS and IV regression results, Choice Reaction Time and Variability. IV defined as proportion of women affected by Marriage Bar by birth cohort (*PropMarBar*)

|  | OLS | | First Stage IV | | Reduced Form | | Second Stage IV | |
| --- | --- | --- | --- | --- | --- | --- | --- | --- |
|  | (1) | (2) | (3) | (4) | (5) | (6) | (7) | (8) |
| Dependent Variable | -ln(CRT) | -ln(CRT_VAR) | RetDur | RetDur | -ln(CRT) | -ln(CRT_VAR) | -ln(CRT) | -ln(CRT_VAR) |
| *PropMarBar* | –– | –– | 13.27* | 13.27* | 0.283* | 0.250 | –– | –– |
|  | –– | –– | (2.6) | (2.6) | (2.3) | (0.9) | –– | –– |
| *RetDur* | -0.00103^*^ | -0.00288^*^ | –– | –– | –– | –– | 0.0213† | 0.0189 |
|  | (-2.1) | (-2.5) | –– | –– | –– | –– | (1.7) | (0.8) |
| *Age* | -0.00779^***^ | -0.0216^***^ | 0.807*** | 0.807*** | -0.0116*** | -0.0268*** | -0.0289* | -0.0421† |
|  | (-9.2) | (-10.8) | (13.3) | (13.3) | (-8.1) | (-7.9) | (-2.4) | (-1.9) |
| *School* | 0.00475^†^ | 0.0163^**^ | -0.546*** | -0.546*** | 0.00521* | 0.0178** | 0.0169* | 0.0281* |
|  | (1.9) | (2.8) | (-5.2) | (-5.2) | (2.1) | (3.0) | (2.2) | (2.0) |
| *NoBooks* | -0.0459^***^ | -0.0881^**^ | 0.453 | 0.453 | -0.0464*** | -0.0894** | -0.0560** | -0.0980** |
|  | (-3.3) | (-2.7) | (0.8) | (0.8) | (-3.3) | (-2.7) | (-2.8) | (-2.7) |
| *PoorHealth* | -0.00604 | 0.0214 | -0.581 | -0.581 | -0.00406 | 0.0244 | 0.00833 | 0.0353 |
|  | (-0.2) | (0.3) | (-0.5) | (-0.5) | (-0.2) | (0.4) | (0.2) | (0.5) |
| *PoorFam* | -0.0166 | -0.0408 | 0.349 | 0.349 | -0.0186 | -0.0434 | -0.0260 | -0.0500 |
|  | (-0.9) | (-1.0) | (0.5) | (0.5) | (-1.0) | (-1.0) | (-1.0) | (-1.1) |
| *MotherNotWork* | -0.00453 | -0.0129 | 1.596** | 1.596** | -0.00729 | -0.0186 | -0.0414 | -0.0487 |
|  | (-0.3) | (-0.4) | (2.8) | (2.8) | (-0.5) | (-0.6) | (-1.5) | (-0.9) |
| *FatherNotWork* | -0.00665 | -0.0368 | -0.0255 | -0.0255 | -0.00560 | -0.0357 | -0.00506 | -0.0353 |
|  | (-0.3) | (-0.6) | (-0.02) | (-0.02) | (-0.2) | (-0.6) | (-0.1) | (-0.5) |
| *Constant* | -5.727^***^ | -3.400^***^ | -37.48*** | -37.48*** | -5.526*** | -3.134*** | -4.726*** | -2.427* |
|  | (-88.8) | (-22.3) | (-9.8) | (-9.8) | (-60.9) | (-14.6) | (-8.2) | (-2.3) |
|  |  |  |  |  |  |  |  |  |
| *R^2^ (%)* | 7.9 | 10.3 | 32.4 | 32.4 | 7.9 | 10.1 | –– | –– |
| *N* | 2,383 | 2,383 | 2,383 | 2,383 | 2,383 | 2,383 | 2,383 | 2,383 |
| First Stage IV Statistics | | | | | | | | |
| *F-statistics* | –– | –– | 6.51 | 6.51 | –– | –– | –– | –– |
| *Stock-Yogo Weak Identification Test Critical Values:* | | | | | | | | |
| *10% maximal IV size* | –– | –– | 16.38 | 16.38 | –– | –– | –– | –– |
| *15% maximal IV size* | –– | –– | 8.96 | 8.96 | –– | –– | –– | –– |
| *20% maximal IV size* | –– | –– | 6.66 | 6.66 | –– | –– | –– | –– |
| *25% maximal IV size* | –– | –– | 5.53 | 5.53 | –– | –– | –– | –– |
| Hausman test (H_0_: RetDur is exogenous) | | | | | | | | |
| *χ2* | –– | –– | –– | –– | –– | –– | 5.9 | 0.99 |
| *P-value* | –– | –– | –– | –– | –– | –– | 0.01 | 0.32 |
| *OLS or IV?* | –– | –– | –– | –– | –– | –– | IV | OLS |
| *Notes*: t statistics in parentheses; † p<.10, * p<.05, ** p<.01, *** p<.001. Abbreviations: CRT: Choice Reaction Time. CRT_VAR: CRT Variability | | | | | | | | |

**Table S7** Wald test for robustness of retirement duration effects. Coefficient of RetDur

|  | (1) | (2) | (3) | (4) |
| --- | --- | --- | --- | --- |
| Dependent Variable | -ln(CTT1) | -ln(CTT2) | -ln(CRT) | -ln(CRT_VAR) |
|  |  |  |  |  |
| (1) Baseline | -0.00193^***^ | -0.00142^**^ | -0.00103^*^ | -0.00288^*^ |
| (2) Exclude aged 80+ | -0.00182^**^ | -0.00157^**^ | -0.00116^*^ | -0.00261^*^ |
| χ2 | 0.14 | 0.65 | 0.19 | 0.16 |
| p-value | 0.71 | 0.42 | 0.67 | 0.69 |
| (3) Exclude aged 70+ | -0.00246^**^ | -0.00202^**^ | -0.00172^**^ | -0.00374^*^ |
| χ2 | 0.53 | 1.42 | 1.51 | 0.41 |
| p-value | 0.47 | 0.23 | 0.22 | 0.52 |
| (4) Exclude home assessment | -0.00161^*^ | -0.00145^**^ | -0.000862 | -0.00217^†^ |
| χ2 | 0.73 | 0.01 | 0.28 | 0.89 |
| p-value | 0.39 | 0.92 | 0.60 | 0.35 |
| (5) Exclude unemployed | -0.00194^***^ | -0.00145^**^ | -0.00104^*^ | -0.00262^*^ |
| χ2 | 0.07 | 0.27 | 0.06 | 1.34 |
| p-value | 0.79 | 0.60 | 0.81 | 0.25 |
| (6) Exclude sick/disabled | -0.00145^*^ | -0.00120^**^ | -0.000937^†^ | -0.00269^*^ |
| χ2 | 4.97 | 3.21 | 1.11 | 0.65 |
| p-value | 0.03 | 0.07 | 0.29 | 0.42 |
| (7) Retired if RetDur > 1 year | -0.00193^***^ | -0.00142^**^ | -0.00102^*^ | -0.00286^*^ |
| χ2 | 0.40 | 0.10 | 0.41 | 0.10 |
| p-value | 0.52 | 0.75 | 0.52 | 0.75 |
| (8) Add Age^2^ | -0.00149^*^ | -0.00128^**^ | -0.000679 | -0.00205^†^ |
| χ2 | 13.37 | 2.96 | 7.96 | 10.80 |
| p-value | <0.001 | 0.09 | 0.005 | 0.001 |
| (9) Add Age^2^ and Age^3^ | -0.00150^**^ | -0.00126^**^ | -0.000749 | -0.00216^†^ |
| χ2 | 8.93 | 2.58 | 6.25 | 9.41 |
| p-value | <0.001 | 0.11 | 0.01 | 0.002 |
| *Notes*: See text for explanation of test.  t statistics in parentheses; † p<.10, * p<.05, ** p<.01, *** p<.001. Abbreviations: CTT1: Colour Trail Task 1. CTT2: Colour Trail Task 2. CRT: Choice Reaction Time. CRT_VAR: CRT Variability | | | | |

**Table S8** OLS regression results, Colour Trail Task 1 and 2. Fertility variables are included as controls

|  | (1) | (2) | (3) | (4) | (5) | (6) | (7) | (8) |
| --- | --- | --- | --- | --- | --- | --- | --- | --- |
|  | -ln(CTT1) | -ln(CTT1) | -ln(CTT1) | -ln(CTT1) | -ln(CTT2) | -ln(CTT2) | -ln(CTT2) | -ln(CTT2) |
| RetDur | -0.00211^***^ | -0.00229^***^ | -0.00231^***^ | -0.00231^***^ | -0.00155^***^ | -0.00161^***^ | -0.00161^***^ | -0.00161^***^ |
|  | (-3.7) | (-4.0) | (-4.0) | (-4.0) | (-3.5) | (-3.6) | (-3.6) | (-3.6) |
| Age | -0.0207^***^ | -0.0210^***^ | -0.0220^***^ | -0.0224^***^ | -0.0164^***^ | -0.0165^***^ | -0.0165^***^ | -0.0164^***^ |
|  | (-20.6) | (-20.8) | (-15.5) | (-15.4) | (-21.0) | (-21.0) | (-14.9) | (-14.6) |
| School | 0.0124^***^ | 0.0129^***^ | 0.0133^***^ | 0.0137^***^ | 0.0142^***^ | 0.0144^***^ | 0.0143^***^ | 0.0142^***^ |
|  | (4.1) | (4.3) | (4.4) | (4.5) | (6.2) | (6.2) | (6.2) | (6.1) |
| NoBooks | -0.0572^***^ | -0.0572^***^ | -0.0570^***^ | -0.0571^***^ | -0.0825^***^ | -0.0826^***^ | -0.0825^***^ | -0.0825^***^ |
|  | (-3.4) | (-3.4) | (-3.4) | (-3.4) | (-6.4) | (-6.4) | (-6.4) | (-6.4) |
| PoorHealth | -0.0629^*^ | -0.0625^*^ | -0.0631^*^ | -0.0634^*^ | -0.0435^†^ | -0.0433^†^ | -0.0432^†^ | -0.0432^†^ |
|  | (-2.0) | (-2.0) | (-2.0) | (-2.0) | (-1.8) | (-1.8) | (-1.8) | (-1.8) |
| PoorFam | -0.00654 | -0.00655 | -0.00727 | -0.00733 | -0.0149 | -0.0149 | -0.0147 | -0.0147 |
|  | (-0.3) | (-0.3) | (-0.3) | (-0.3) | (-0.9) | (-0.9) | (-0.9) | (-0.9) |
| MotherNotWork | -0.00457 | -0.00398 | -0.00368 | -0.00365 | -0.0158 | -0.0156 | -0.0157 | -0.0157 |
|  | (-0.3) | (-0.2) | (-0.2) | (-0.2) | (-1.3) | (-1.2) | (-1.2) | (-1.2) |
| FatherNotWork | -0.0303 | -0.0312 | -0.0305 | -0.0303 | -0.0225 | -0.0227 | -0.0243 | -0.0243 |
|  | (-1.0) | (-1.0) | (-1.0) | (-1.0) | (-1.0) | (-1.0) | (-1.0) | (-1.0) |
| HasChild | 0.0790^***^ | 0.0324 | -0.0163 | -0.0365 | 0.0558^**^ | 0.0419^†^ | 0.0417 | 0.0453 |
|  | (3.4) | (1.1) | (-0.3) | (-0.6) | (3.1) | (1.9) | (1.0) | (1.0) |
| NumChild | –– | 0.0137^**^ | 0.0151^**^ | 0.00977 | –– | 0.00410 | 0.00419 | 0.00513 |
|  | –– | (2.8) | (2.9) | (1.4) | –– | (1.1) | (1.1) | (1.0) |
| AgeYoungChild | –– | –– | 0.00137 | -0.000540 | –– | –– | -0.00000790 | 0.000329 |
|  | –– | –– | (1.0) | (-0.3) | –– | –– | (-0.008) | (0.2) |
| AgeOldChild | –– | –– | –– | 0.00253 | –– | –– | –– | -0.000448 |
|  | –– | –– | –– | (1.1) | –– | –– | –– | (-0.3) |
| Constant | -2.679^***^ | -2.664^***^ | -2.600^***^ | -2.583^***^ | -3.693^***^ | -3.689^***^ | -3.689^***^ | -3.692^***^ |
|  | (-32.2) | (-32.0) | (-25.0) | (-24.6) | (-57.5) | (-57.3) | (-45.9) | (-45.5) |
| N | 2,519 | 2,519 | 2,519 | 2,519 | 2,481 | 2,481 | 2,481 | 2,481 |
| R^2^ (%) | 26.6 | 26.8 | 26.8 | 26.9 | 29.7 | 29.7 | 29.7 | 29.7 |
| Wald Test for Comparison of RetDur with Baseline RetDur | | | | | | | | |
| Baseline RetDur | -0.00193^***^ | -0.00193^***^ | -0.00193^***^ | -0.00193^***^ | -0.00142^**^ | -0.00142^**^ | -0.00142^**^ | -0.00142^**^ |
| χ2 | 7.45 | 8.21 | 8.45 | 8.44 | 8.30 | 6.78 | 6.50 | 6.49 |
| p-value | 0.006 | 0.004 | 0.004 | 0.004 | 0.004 | 0.009 | 0.011 | 0.011 |
| *Notes*: t statistics in parentheses; † p<.10, * p<.05, ** p<.01, *** p<.001. Abbreviations: : CTT1: Colour Trail Task 1. CTT2: Colour Trail Task 2 | | | | | | | | |

**Table S9** OLS regression results, Choice Reaction Time and Variability. Fertility variables are included as controls

|  | (1) | (2) | (3) | (4) | (5) | (6) | (7) | (8) |
| --- | --- | --- | --- | --- | --- | --- | --- | --- |
|  | -ln(CRT) | -ln(CRT) | -ln(CRT) | -ln(CRT) | -ln(CRT_VAR) | -ln(CRT_VAR) | -ln(CRT_VAR) | -ln(CRT_VAR) |
| *RetDur* | -0.00111^*^ | -0.00113^*^ | -0.00114^*^ | -0.00114^*^ | -0.00299^**^ | -0.00292^*^ | -0.00292^*^ | -0.00293^*^ |
|  | (-2.3) | (-2.3) | (-2.3) | (-2.3) | (-2.6) | (-2.5) | (-2.5) | (-2.5) |
| *Age* | -0.00761^***^ | -0.00763^***^ | -0.00855^***^ | -0.00871^***^ | -0.0213^***^ | -0.0212^***^ | -0.0210^***^ | -0.0214^***^ |
|  | (-8.9) | (-8.9) | (-7.1) | (-7.1) | (-10.6) | (-10.4) | (-7.4) | (-7.3) |
| *School* | 0.00528^*^ | 0.00533^*^ | 0.00566^*^ | 0.00586^*^ | 0.0171^**^ | 0.0169^**^ | 0.0168^**^ | 0.0173^**^ |
|  | (2.1) | (2.1) | (2.2) | (2.3) | (2.9) | (2.9) | (2.8) | (2.9) |
| *NoBooks* | -0.0466^***^ | -0.0466^***^ | -0.0466^***^ | -0.0466^***^ | -0.0892^**^ | -0.0892^**^ | -0.0890^**^ | -0.0890^**^ |
|  | (-3.4) | (-3.4) | (-3.4) | (-3.4) | (-2.7) | (-2.7) | (-2.7) | (-2.7) |
| *PoorHealth* | -0.00372 | -0.00372 | -0.00419 | -0.00446 | 0.0246 | 0.0246 | 0.0248 | 0.0241 |
|  | (-0.1) | (-0.1) | (-0.2) | (-0.2) | (0.4) | (0.4) | (0.4) | (0.4) |
| *PoorFam* | -0.0149 | -0.0149 | -0.0153 | -0.0154 | -0.0383 | -0.0382 | -0.0378 | -0.0381 |
|  | (-0.8) | (-0.8) | (-0.8) | (-0.9) | (-0.9) | (-0.9) | (-0.9) | (-0.9) |
| *MotherNotWork* | -0.00470 | -0.00464 | -0.00444 | -0.00440 | -0.0131 | -0.0133 | -0.0136 | -0.0135 |
|  | (-0.3) | (-0.3) | (-0.3) | (-0.3) | (-0.4) | (-0.4) | (-0.4) | (-0.4) |
| *FatherNotWork* | -0.00663 | -0.00668 | -0.00741 | -0.00732 | -0.0368 | -0.0366 | -0.0400 | -0.0398 |
|  | (-0.3) | (-0.3) | (-0.3) | (-0.3) | (-0.6) | (-0.6) | (-0.7) | (-0.7) |
| *HasChild* | 0.0366^†^ | 0.0324 | -0.0109 | -0.0203 | 0.0519 | 0.0677 | 0.0761 | 0.0522 |
|  | (1.9) | (1.3) | (-0.2) | (-0.4) | (1.1) | (1.2) | (0.7) | (0.5) |
| *NumChild* | –– | 0.00126 | 0.00258 | 0.000125 | –– | -0.00468 | -0.00475 | -0.0110 |
|  | –– | (0.3) | (0.6) | (0.02) | –– | (-0.5) | (-0.5) | (-0.8) |
| *AgeYoungChild* | –– | –– | 0.00122 | 0.000344 | –– | –– | -0.000267 | -0.00250 |
|  | –– | –– | (1.1) | (0.2) | –– | –– | (-0.1) | (-0.6) |
| *AgeOldChild* | –– | –– | –– | 0.00117 | –– | –– | –– | 0.00298 |
|  | –– | –– | –– | (0.6) | –– | –– | –– | (0.7) |
| *Constant* | -5.778^***^ | -5.776^***^ | -5.719^***^ | -5.711^***^ | -3.471^***^ | -3.476^***^ | -3.488^***^ | -3.466^***^ |
|  | (-82.6) | (-82.4) | (-65.4) | (-64.6) | (-21.0) | (-20.9) | (-16.8) | (-16.5) |
| *N* | 2,283 | 2,283 | 2,283 | 2,283 | 2,283 | 2,283 | 2,283 | 2,283 |
| *R^2^ (%)* | 8.0 | 8.0 | 8.1 | 8.1 | 10.4 | 10.4 | 10.4 | 10.4 |
| *Wald Test for Comparison of RetDur with Baseline RetDur* | | | | | | | | |
| Baseline RetDur | -0.00103^*^ | -0.00103^*^ | -0.00103^*^ | -0.00103^*^ | -0.00288^*^ | -0.00288^*^ | -0.00288^*^ | -0.00288^*^ |
| χ2 | 2.93 | 1.61 | 1.89 | 1.92 | 1.18 | 0.07 | 0.06 | 0.06 |
| p-value | 0.087 | 0.200 | 0.170 | 0.166 | 0.277 | 0.796 | 0.811 | 0.801 |
| *Notes*: t statistics in parentheses; † p<.10, * p<.05, ** p<.01, *** p<.001. Abbreviations: CRT: Choice Reaction Time. CRT_VAR: CRT Variability | | | | | | | | |

**Table S10** Proportion engaging into volunteering activity at least once per week by age at retirement

| Age X | Retired before age X | Retired at or after age X | Test of Equality in Proportions (p value) |
| --- | --- | --- | --- |
| 30 | 21.6%  (N=56) | 19.4%  (N=251) | 0.41 |
| 35 | 22.3%  (N=75) | 19.1%  (N=232) | 0.20 |
| 40 | 21.3%  (N=81) | 19.3%  (N=226) | 0.39 |
| *Note*: Results are for women who are retired at TILDA Wave 3 data collection and who have ever been married | | | |

**Table S11** OLS regression results. RetDur defined as “Time elapsed since marriage” for 39 women

|  | (1) | (2) | (3) | (4) |
| --- | --- | --- | --- | --- |
|  | -ln(CTT1) | -ln(CTT2) | -ln(CRT) | -ln(CRT_VAR) |
| *RetDur* | -0.00167** | -0.00135** | -0.00130** | -0.00289** |
|  | (-3.0) | (-3.1) | (-2.8) | (-2.6) |
| *Age* | -0.0213*** | -0.0167*** | -0.00750*** | -0.0215*** |
|  | (-21.3) | (-21.6) | (-8.8) | (-10.7) |
| *School* | 0.0113*** | 0.0134*** | 0.00460† | 0.0163** |
|  | (3.8) | (5.9) | (1.9) | (2.8) |
| *NoBooks* | -0.0557*** | -0.0815*** | -0.0457** | -0.0879** |
|  | (-3.3) | (-6.3) | (-3.3) | (-2.7) |
| *PoorHealth* | -0.0681* | -0.0473† | -0.00583 | 0.0222 |
|  | (-2.2) | (-2.0) | (-0.2) | (0.4) |
| *PoorFam* | -0.00952 | -0.0171 | -0.0165 | -0.0408 |
|  | (-0.4) | (-1.0) | (-0.9) | (-1.0) |
| *MotherNotWork* | -0.00427 | -0.0154 | -0.00394 | -0.0126 |
|  | (-0.3) | (-1.2) | (-0.3) | (-0.4) |
| *FatherNotWork* | -0.0317 | -0.0231 | -0.00728 | -0.0382 |
|  | (-1.0) | (-1.0) | (-0.3) | (-0.6) |
| *Constant* | -2.558*** | -3.614*** | -5.742*** | -3.406*** |
|  | (-33.5) | (-61.2) | (-89.0) | (-22.3) |
|  |  |  |  |  |
| *R^2^ (%)* | 26.2 | 29.4 | 8.0 | 10.3 |
| *N* | 2,519 | 2,481 | 2,383 | 2,383 |
|  |  |  |  |  |
| *Wald Test for Comparison of RetDur with Baseline RetDur* | | | | |
| Baseline RetDur | -0.00193^***^ | -0.00142^**^ | -0.00103^*^ | -0.00288^*^ |
| χ2 | 2.24 | 0.20 | 1.12 | 0.00 |
| p-value | 0.13 | 0.65 | 0.29 | 0.97 |
| *Notes*: t statistics in parentheses; † p<.10, * p<.05, ** p<.01, *** p<.001. RetDur = time elapsed since marriage for women who left a job because of the marriage bar and returned to work after 20+ years (N=39); = time elapsed since last job ended for the remaining of the sample.  Abbreviations: CTT1: Colour Trail Task 1. CTT2: Colour Trail Task 2. CRT: Choice Reaction Time. CRT_VAR: CRT Variability | | | | |

## Table S12 OLS regression results. RetDur interacted with NonManual occupation

|  | (1) | (2) | (3) | (4) | (5) | (6) | (7) | (8) | (9) | (10) | (11) | (12) |
| --- | --- | --- | --- | --- | --- | --- | --- | --- | --- | --- | --- | --- |
|  | -ln(CTT1) | -ln(CTT2) | -ln(CRT) | -ln(CRT_VAR) | -ln(CTT1) | -ln(CTT2) | -ln(CRT) | -ln(CRT_VAR) | -ln(CTT1) | -ln(CTT2) | -ln(CRT) | -ln(CRT_VAR) |
| *RetDur* | -0.00408^***^ | -0.00356^***^ | -0.00188^**^ | -0.00551^***^ | -0.00175^**^ | -0.00143^**^ | -0.00121^*^ | -0.00309^*^ | -0.00179^**^ | -0.00179^**^ | -0.00118^*^ | -0.00324^**^ |
|  | (-5.1) | (-5.7) | (-2.8) | (-3.4) | (-2.9) | (-3.0) | (-2.4) | (-2.6) | (-3.0) | (-3.0) | (-2.4) | (-2.8) |
| *NonManual*RetDur* | 0.00361^***^ | 0.00344^***^ | 0.00110 | 0.00347^*^ | -0.000395 | 0.000416 | 0.000277 | -0.00106 | 0.00414 | 0.00414 | -0.00300 | 0.00173 |
|  | (4.3) | (5.2) | (1.5) | (2.0) | (-0.4) | (0.5) | (0.3) | (-0.5) | (0.9) | (0.9) | (-0.7) | (0.2) |
| *Age* | -0.0214^***^ | -0.0172^***^ | -0.00747^***^ | -0.0212^***^ | -0.0209^***^ | -0.0168^***^ | -0.00740^***^ | -0.0206^***^ | -0.0211^***^ | -0.0211^***^ | -0.00727^***^ | -0.0209^***^ |
|  | (-20.5) | (-21.4) | (-8.6) | (-10.2) | (-19.7) | (-20.6) | (-8.4) | (-9.8) | (-20.1) | (-20.1) | (-8.4) | (-10.0) |
| *School* | 0.00811^*^ | 0.0107^***^ | 0.00501^†^ | 0.0156^*^ | 0.0115^***^ | 0.0132^***^ | 0.00571^*^ | 0.0193^**^ | 0.0110^***^ | 0.0110^***^ | 0.00605^*^ | 0.0184^**^ |
|  | (2.6) | (4.4) | (1.9) | (2.5) | (3.6) | (5.4) | (2.2) | (3.1) | (3.6) | (3.6) | (2.4) | (3.1) |
| *NoBooks* | -0.0470^**^ | -0.0698^***^ | -0.0347^*^ | -0.0643^†^ | -0.0532^**^ | -0.0759^***^ | -0.0363^*^ | -0.0702^*^ | -0.0528^**^ | -0.0528^**^ | -0.0367^**^ | -0.0696^*^ |
|  | (-2.7) | (-5.3) | (-2.5) | (-1.9) | (-3.1) | (-5.7) | (-2.6) | (-2.1) | (-3.0) | (-3.0) | (-2.6) | (-2.1) |
| *PoorHealth* | -0.0572^†^ | -0.0484^†^ | 0.0143 | 0.0375 | -0.0530 | -0.0452^†^ | 0.0156 | 0.0442 | -0.0539^†^ | -0.0539^†^ | 0.0164 | 0.0425 |
|  | (-1.8) | (-2.0) | (0.5) | (0.6) | (-1.6) | (-1.8) | (0.6) | (0.7) | (-1.7) | (-1.7) | (0.6) | (0.7) |
| *PoorFam* | -0.00311 | -0.0142 | -0.0210 | -0.0450 | -0.0100 | -0.0212 | -0.0232 | -0.0520 | -0.0101 | -0.0101 | -0.0233 | -0.0521 |
|  | (-0.1) | (-0.8) | (-1.2) | (-1.0) | (-0.4) | (-1.2) | (-1.3) | (-1.2) | (-0.5) | (-0.5) | (-1.3) | (-1.2) |
| *MotherNotWork* | -0.00268 | -0.0149 | -0.00759 | -0.0173 | -0.00190 | -0.0149 | -0.00755 | -0.0167 | -0.00223 | -0.00223 | -0.00728 | -0.0171 |
|  | (-0.2) | (-1.2) | (-0.5) | (-0.5) | (-0.1) | (-1.1) | (-0.5) | (-0.5) | (-0.1) | (-0.1) | (-0.5) | (-0.5) |
| *FatherNotWork* | -0.0261 | -0.0210 | 0.00206 | -0.0316 | -0.0254 | -0.0215 | 0.00220 | -0.0288 | -0.0264 | -0.0264 | 0.00252 | -0.0301 |
|  | (-0.8) | (-0.9) | (0.08) | (-0.5) | (-0.8) | (-0.9) | (0.09) | (-0.5) | (-0.8) | (-0.8) | (0.10) | (-0.5) |
| *Constant* | -2.520^***^ | -3.557^***^ | -5.750^***^ | -3.414^***^ | -2.592^***^ | -3.607^***^ | -5.763^***^ | -3.497^***^ | -2.574^***^ | -2.574^***^ | -5.775^***^ | -3.470^***^ |
|  | (-31.3) | (-57.6) | (-86.3) | (-21.4) | (-31.4) | (-57.0) | (-84.8) | (-21.5) | (-32.1) | (-32.1) | (-87.5) | (-22.0) |
| *N* | 2,365 | 2,330 | 2,234 | 2,234 | 2,365 | 2,330 | 2,234 | 2,234 | 2,365 | 2,365 | 2,234 | 2,234 |
| *R^2^ (%)* | 26.3 | 30.5 | 8.1 | 10.7 | 25.8 | 29.7 | 8.0 | 10.5 | 25.8 | 25.8 | 8.0 | 10.5 |
|  |  |  |  |  |  |  |  |  |  |  |  |  |
| Wald Test of NonManual*RetDur Interaction | | | | | | | | | | | | |
| F test | 18.2*** | 27.5*** | 2.4 | 4.1* | 0.1 | 0.3 | 0.1 | 0.3 | 0.8 | 1.3 | 0.6 | 0.0 |
| *Notes*: t statistics in parentheses: † p<.10, * p<.05, ** p<.01, *** p<.001. Abbreviations: CTT1: Colour Trail Task 1. CTT2: Colour Trail Task 2. CRT: Choice Reaction Time. CRT_VAR: CRT Variabilit**y**Non-manual group includes: professional, managerial & non-manual in (1) to (4); professional & managerial in (5) to (8); professional only in (9) to (12)Manual group includes: skilled manual, semi-skilled and unskilled in (1) to (4); non-manual, skilled manual, semi-skilled and unskilled in (5) to (8); managerial, non-manual, skilled manual, semi-skilled and unskilled in (9) to (12) | | | | | | | | | | | | |

##### **Table S13** OLS regression results. RetDur interacted with PartTime

|  | (1) | (2) | (3) | (4) | | (5) | (6) | (7) | (8) | (9) | (10) | (11) | (12) | |
| --- | --- | --- | --- | --- | --- | --- | --- | --- | --- | --- | --- | --- | --- | --- |
|  | -ln(CTT1) | -ln(CTT2) | -ln(CRT) | -ln(CRT_VAR) | | -ln(CTT1) | -ln(CTT2) | -ln(CRT) | -ln(CRT_VAR) | -ln(CTT1) | -ln(CTT2) | -ln(CRT) | -ln(CRT_VAR) | |
|  |  |  |  |  |  | |  |  |  |  |  |  |  | |
| *RetDur* | -0.00121 | -0.00117^*^ | -0.00128^*^ | -0.00271^†^ | -0.00127 | | -0.00122^*^ | -0.00128^*^ | -0.00281^†^ | -0.00124 | -0.00114^†^ | -0.00113^†^ | -0.00257^†^ | |
|  | (-1.5) | (-2.0) | (-2.0) | (-1.8) | (-1.6) | | (-2.1) | (-2.0) | (-1.8) | (-1.6) | (-2.0) | (-1.8) | (-1.7) | |
| *PartTime*RetDur* | -0.00113 | -0.0000831 | 0.00239^*^ | 0.00305 | -0.000514 | | 0.000351 | 0.00221^*^ | 0.00389 | -0.000777 | -0.000438 | 0.000781 | 0.00142 | |
|  | (-0.8) | (-0.08) | (2.1) | (1.1) | (-0.4) | | (0.4) | (2.1) | (1.5) | (-0.6) | (-0.5) | (0.8) | (0.6) | |
| *Age* | -0.0218^***^ | -0.0171^***^ | -0.00798^***^ | -0.0223^***^ | -0.0218^***^ | | -0.0171^***^ | -0.00805^***^ | -0.0225^***^ | -0.0217^***^ | -0.0171^***^ | -0.00798^***^ | -0.0224^***^ | |
|  | (-18.0) | (-18.9) | (-7.9) | (-9.4) | (-17.9) | | (-18.9) | (-8.0) | (-9.4) | (-17.8) | (-18.7) | (-7.9) | (-9.3) | |
| *School* | 0.00965^**^ | 0.0119^***^ | 0.00474^†^ | 0.0181^**^ | 0.00978^**^ | | 0.0119^***^ | 0.00459^†^ | 0.0181^**^ | 0.00977^**^ | 0.0119^***^ | 0.00437 | 0.0177^**^ | |
|  | (3.0) | (4.9) | (1.8) | (2.9) | (3.0) | | (4.9) | (1.7) | (2.9) | (3.0) | (4.9) | (1.6) | (2.8) | |
| *NoBooks* | -0.0479^*^ | -0.0798^***^ | -0.0559^***^ | -0.0890^*^ | -0.0481^*^ | | -0.0799^***^ | -0.0557^***^ | -0.0892^*^ | -0.0479^*^ | -0.0797^***^ | -0.0551^***^ | -0.0881^*^ | |
|  | (-2.5) | (-5.7) | (-3.6) | (-2.5) | (-2.6) | | (-5.7) | (-3.6) | (-2.5) | (-2.5) | (-5.7) | (-3.6) | (-2.4) | |
| *PoorHealth* | -0.0575 | -0.0335 | 0.00917 | 0.0905 | -0.0576 | | -0.0338 | 0.00882 | 0.0890 | -0.0575 | -0.0333 | 0.0104 | 0.0917 | |
|  | (-1.6) | (-1.2) | (0.3) | (1.3) | (-1.6) | | (-1.2) | (0.3) | (1.2) | (-1.6) | (-1.2) | (0.3) | (1.3) | |
| *PoorFam* | -0.0179 | -0.0138 | -0.0195 | -0.0397 | -0.0182 | | -0.0141 | -0.0196 | -0.0402 | -0.0176 | -0.0134 | -0.0193 | -0.0398 | |
|  | (-0.7) | (-0.8) | (-1.0) | (-0.8) | (-0.7) | | (-0.8) | (-1.0) | (-0.8) | (-0.7) | (-0.7) | (-1.0) | (-0.8) | |
| *MotherNotWork* | -0.0124 | -0.0261^†^ | -0.0172 | -0.0210 | -0.0124 | | -0.0261^†^ | -0.0168 | -0.0203 | -0.0125 | -0.0261^†^ | -0.0173 | -0.0210 | |
|  | (-0.7) | (-1.9) | (-1.1) | (-0.6) | (-0.7) | | (-1.9) | (-1.1) | (-0.6) | (-0.7) | (-1.9) | (-1.1) | (-0.6) | |
| *FatherNotWork* | -0.00995 | -0.0159 | -0.0154 | -0.0606 | -0.0100 | | -0.0159 | -0.0157 | -0.0610 | -0.00984 | -0.0158 | -0.0154 | -0.0607 | |
|  | (-0.3) | (-0.6) | (-0.6) | (-0.9) | (-0.3) | | (-0.6) | (-0.6) | (-1.0) | (-0.3) | (-0.6) | (-0.6) | (-0.9) | |
| *Constant* | -2.500^***^ | -3.560^***^ | -5.703^***^ | -3.364^***^ | -2.502^***^ | | -3.559^***^ | -5.697^***^ | -3.354^***^ | -2.505^***^ | -3.562^***^ | -5.698^***^ | -3.355^***^ | |
|  | (-27.5) | (-52.6) | (-75.6) | (-18.9) | (-27.5) | | (-52.5) | (-75.5) | (-18.8) | (-27.5) | (-52.4) | (-75.2) | (-18.8) | |
| *N* | 2,057 | 2,025 | 1,954 | 1,954 | 2,057 | | 2,025 | 1,954 | 1,954 | 2,056 | 2,024 | 1,953 | 1,953 | |
| *R^2^ (%)* | 26.1% | 30.8% | 8.9% | 11.1% | 26.1% | | 30.8% | 8.9% | 11.1% | 26.1% | 30.8% | 8.8% | 11.1% | |
|  |  |  |  |  |  | |  |  |  |  |  |  |  | |
| Wald Test of PartTime*RetDur Interaction | | | | | | | | | | | | | | |
| F test | 0.40 | 0.01 | 4.27* | 1.24 | 0.17 | | 0.13 | 4.23* | 2.36 | 0.42 | 0.23 | 0.59 | 0.35 | |
| *Notes*: t statistics in parentheses: † p<.10, * p<.05, ** p<.01, *** p<.001. Abbreviations: CTT1: Colour Trail Task 1. CTT2: Colour Trail Task 2. CRT: Choice Reaction Time. CRT_VAR: CRT VariabilityAverage hours worked per week by part-time group are: 1-20 in (1) to (4); 1-25 in (5) to (8); 1-30 in (9) to (12)Average hours worked per week by full-time group are: 21-40+ in (1) to (4); 26-40+ in (5) to (8); 31-40+ in (9) to (12) | | | | | | | | | | | | | |  |

**Table S14** OLS regression results. RetDur interacted with AgeRet

|  | (1) | (2) | (3) | (4) |
| --- | --- | --- | --- | --- |
|  | -ln(CTT1) | -ln(CTT2) | -ln(CRT) | -ln(CRT_VAR) |
| *RetDur* | -0.0182^***^ | -0.0120^***^ | -0.00743^***^ | -0.0222^***^ |
|  | (-8.1) | (-7.0) | (-3.9) | (-5.0) |
| *AgeRet* | -0.0187^***^ | -0.0137^***^ | -0.00727^***^ | -0.0212^***^ |
|  | (-12.0) | (-11.6) | (-5.5) | (-6.8) |
| *RetDur*AgeRet* | -0.000123^*^ | -0.000144^***^ | -0.0000305 | -0.0000655 |
|  | (-2.5) | (-3.7) | (-0.7) | (-0.7) |
| *School* | 0.0129^***^ | 0.0141^***^ | 0.00389 | 0.0144^†^ |
|  | (3.5) | (5.0) | (1.2) | (1.9) |
| *NoBooks* | -0.0475^*^ | -0.0651^***^ | -0.0432^*^ | -0.0796^†^ |
|  | (-2.3) | (-4.1) | (-2.4) | (-1.9) |
| *PoorHealth* | -0.0813^*^ | -0.0563^†^ | -0.00390 | 0.0240 |
|  | (-2.1) | (-1.9) | (-0.1) | (0.3) |
| *PoorFam* | -0.00571 | -0.0215 | -0.0156 | -0.0563 |
|  | (-0.2) | (-1.1) | (-0.7) | (-1.0) |
| *MotherNotWork* | -0.00376 | -0.0239 | -0.0113 | -0.0378 |
|  | (-0.2) | (-1.5) | (-0.6) | (-0.9) |
| *FatherNotWork* | -0.0678^†^ | -0.0308 | -0.0257 | -0.126^†^ |
|  | (-1.8) | (-1.1) | (-0.8) | (-1.7) |
| *Contant* | -2.714^***^ | -3.786^***^ | -5.746^***^ | -3.370^***^ |
|  | (-25.6) | (-47.1) | (-63.6) | (-15.8) |
| *N* | 1,736 | 1,698 | 1,619 | 1,619 |
| *R*^2^ *(%)* | 22.9 | 25.6 | 6.1 | 8.8 |
|  |  |  |  |  |
| *Wald Test of RetDur*AgeRet Interaction* | | | | |
| F test | 6.2* | 13.8*** | 0.5 | 0.4 |
| *Notes*: t statistics in parentheses; † p<.10, * p<.05, ** p<.01, *** p<.001.  Abbreviations: CTT1: Colour Trail Task 1. CTT2: Colour Trail Task 2. CRT: Choice Reaction Time. CRT_VAR: CRT Variability  Only women currently retired are included in the sample | | | | |

**Fig. S1** Cognitive measures by age**.** Panel (a) shows CTT1, Colour Trail Task 1. Panel (b) shows CTT2, Colour Trail Task 2. Panel(c) shows CRT, Choice Reation Time. Panel (d) shows CRT_VAR, Choice Reation Time Variabiliy. On the right-hand side, each cognitive measures is expressed as a z-score. Solid line is linear fit. Dashed line is linear fit for z-score.


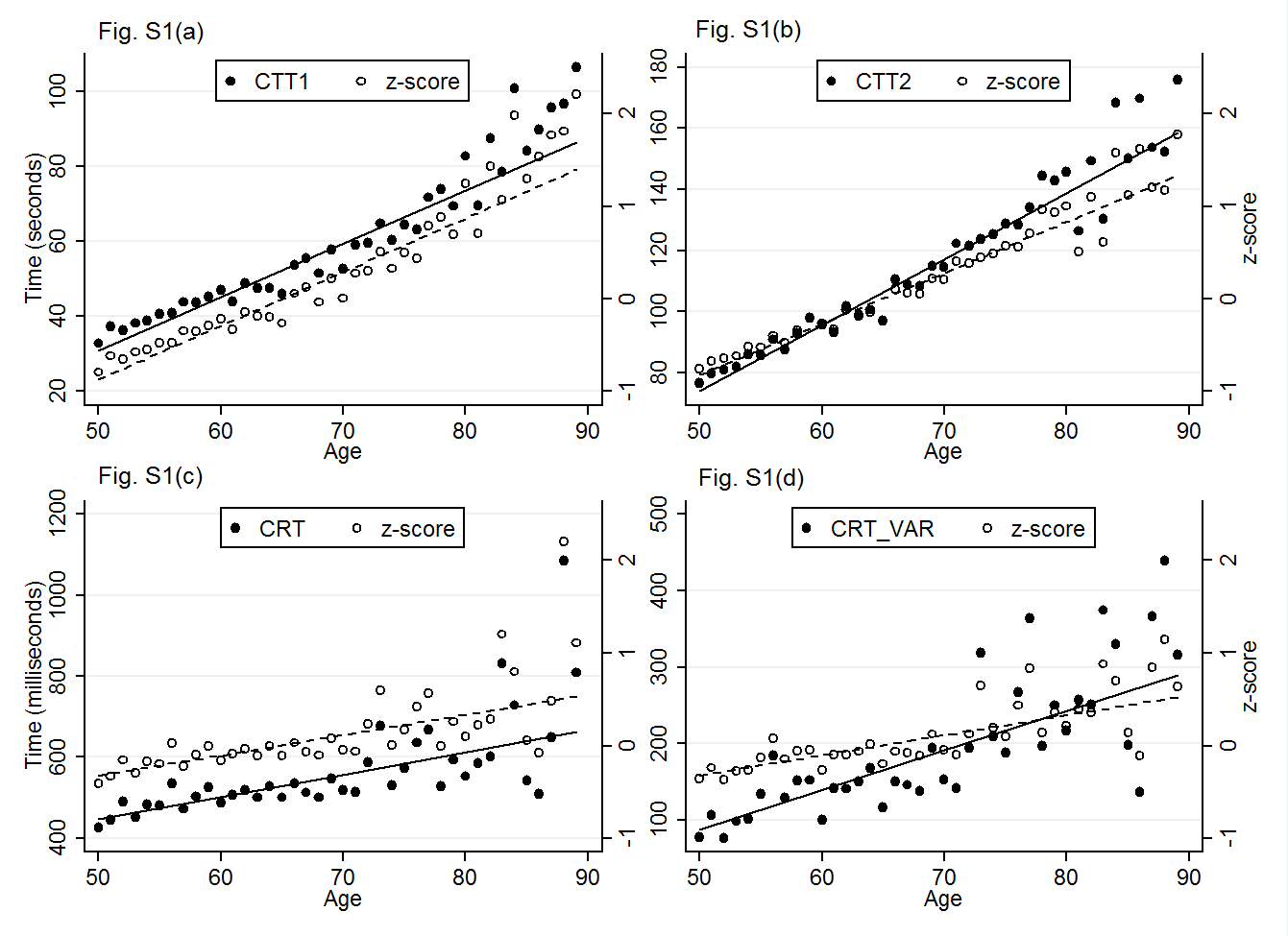


**Fig. S2** Cognitive measures by retirment duration. Panel (a) shows CTT1, Colour Trail Task 1. Panel (b) shows CTT2, Colour Trail Task 2. Panel (c) shows CRT, Choice Reation Time. Panel (d) shows CRT_VAR, Choice Reation Time Variabiliy. On the right-hand side, each cognitive measures is expressed as a z-score. Solid line is linear fit. Dashed line is linear fit for z-score.


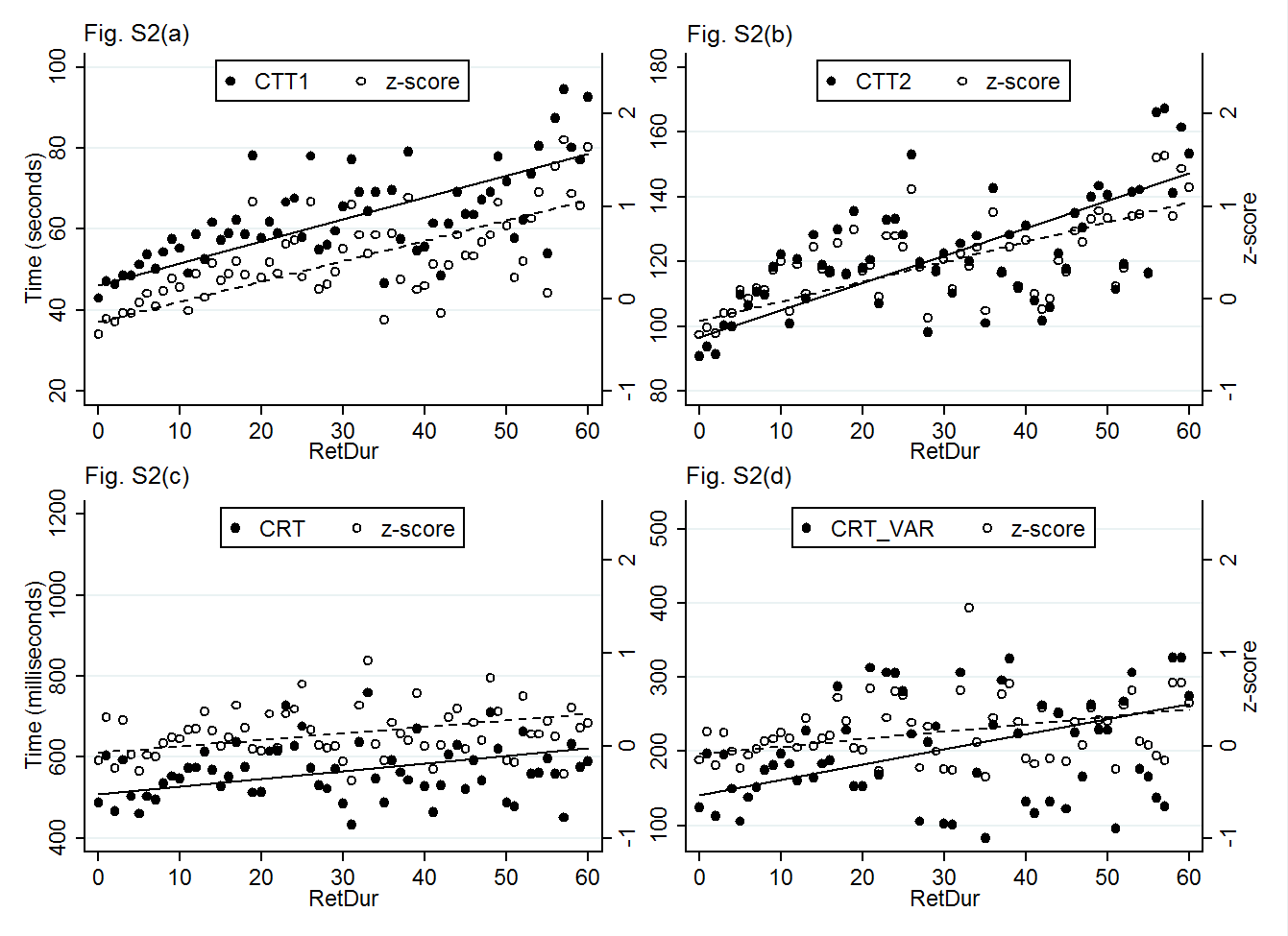

Supplement: Supplementary file 1 — (DOCX 273 kb) [file 13524_2018_682_MOESM1_ESM.docx]
